# Identification of host receptors for viral entry and beyond: a perspective from the spike of SARS-CoV-2

**DOI:** 10.3389/fmicb.2023.1188249

**Published:** 2023-07-25

**Authors:** Xuhua Xia

**Affiliations:** ^1^Department of Biology, University of Ottawa, Ottawa, ON, Canada; ^2^Ottawa Institute of Systems Biology, University of Ottawa, Ottawa, ON, Canada

**Keywords:** receptor-binding protein, host receptor, protein structure, phylogenetics, gene expression, drug target

## Abstract

Identification of the interaction between the host membrane receptor and viral receptor-binding domain (RBD) represents a crucial step for understanding viral pathophysiology and for developing drugs against pathogenic viruses. While all membrane receptors and carbohydrate chains could potentially be used as receptors for viruses, prioritized searches focus typically on membrane receptors that are known to have been used by the relatives of the pathogenic virus, e.g., ACE2 used as a receptor for SARS-CoV is a prioritized candidate receptor for SARS-CoV-2. An ideal receptor protein from a viral perspective is one that is highly expressed in epithelial cell surface of mammalian respiratory or digestive tracts, strongly conserved in evolution so many mammalian species can serve as potential hosts, and functionally important so that its expression cannot be readily downregulated by the host in response to the infection. Experimental confirmation of host receptors includes (1) infection studies with cell cultures/tissues/organs with or without candidate receptor expression, (2) experimental determination of protein structure of the complex between the putative viral RDB and the candidate host receptor, and (3) experiments with mutant candidate receptor or homologues of the candidate receptor in other species. Successful identification of the host receptor opens the door for mechanism-based development of candidate drugs and vaccines and facilitates the inference of what other animal species are vulnerable to the viral pathogen. I illustrate these approaches with research on identification of the receptor and co-factors for SARS-CoV-2.

## 1. Introduction

Many processes are involved in viral infection, including attachment to host cells, entry of host cells, evasion of host defense mechanisms, viral genome replication, transcription and translation within host cells, viral packaging, lysing host cells and initiating a new infection cycle. Among these processes, attaching to and entering the host cell are often the limiting step requiring the viral pathogen to evolve specific adaptation to the host. Once inside the host cell, the cytoplasmic environment for viral genome replication, transcription and translation are similar across diverse mammalian species.

The need and urgency of identifying the host receptor used by viruses are highlighted by the COVID-19 pandemic. What is the host receptor for the spike protein of SARS-CoV-2 (SARS-2S)? Which part of the SARS-2S binds to which part of the host receptor? What are the amino acid residues that interact between the host receptor and the viral receptor-binding protein? Can drugs be developed to block the binding of SARS-2S to the receptor? Will the drug interfere with the normal function of the receptor and cause a strong side effect? What are the normal functions of the host receptor? Are people who express less of this receptor protein less vulnerable to COVID-19 infection? What other mammalian species have similar host receptors that render them vulnerable to SARS-CoV-2 infection? How well can we predict species vulnerability based on receptor protein similarity in sequence and in structure? Answers to these questions contribute to a good understanding of pathophysiology and epidemiology and provide a foundation for drug development. I illustrate the multi-omics approaches to address these questions related to host receptors.

## 2. Identification of host receptor and cofactors

Cell membranes are composed of many single-pass transmembrane receptors. Some of them can be internalized into cells upon ligand binding, and could serve as candidate receptors mediating viral attachment and cell entry. However, instead of screening all of them as candidate receptors, existing biological knowledge can speed up the search of host receptors or cofactors that support or enhance viral attachment and cell entry.

### 2.1. Identification of ACE2 as the host receptor

The first genomic sequence of SARS-CoV-2 was obtained on January 5, 2020, and made public on Jan. 11, 2020 (Wu et al., 2020). Previously, ACE2 was found to be the host receptor of SARS-S (Li et al., 2003; Kuba et al., 2005). The receptor-binding domain of SARS-S alone can bind to ACE2, leading to its internalization together with the host ACE2 (Wang et al., 2008). Because of the similarity in sequence and domain organization between SARS-S and SARS-2S (Zhou P. et al., 2020; Xia, 2021), it is natural to infer that SARS-2S may use the same host receptor ACE2 as SARS-S (Zhou P. et al., 2020).

**Figure 1:**
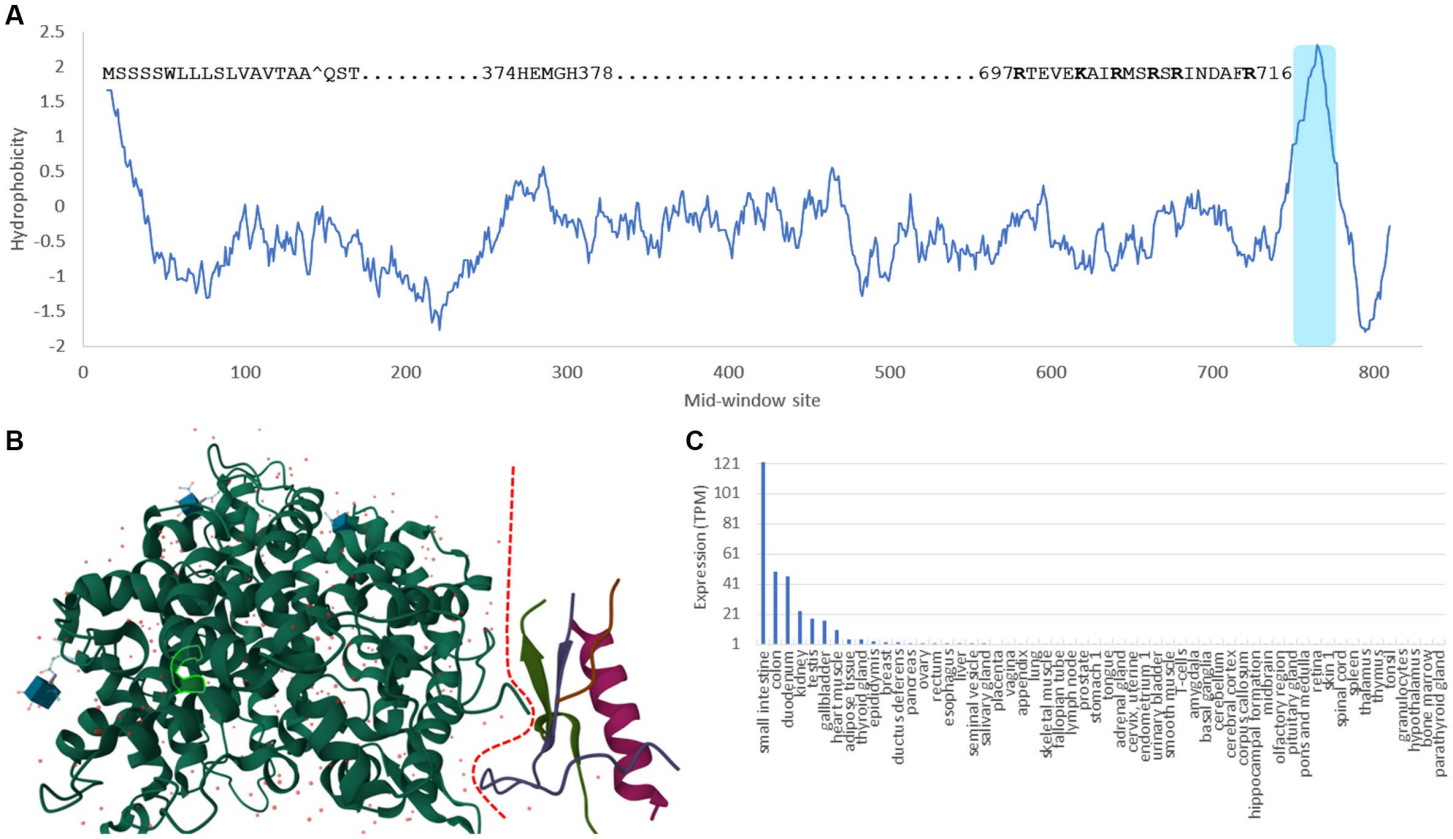
Domains, structure and tissue-specific expression of human ACE2. **(A)** Hydrophobicity plot generated from DAMBE (Xia, 2018b) based on hydrophobicity values in Kyte and Doolittle (1982) along a sliding window of 40 amino acids. The 17-aa signal peptide at the N-terminus, the 5-aa zinc-binding motif, the segment rich in lysine and arginine (KR-rich segment) serving as cleavage sites for TMPRSS2 and HAT proteases (Heurich et al., 2014), and the shaded hydrophobic transmembrane domain are indicated. **(B)** Structure of ACE2 (1R42) (Towler et al., 2004) with the dashed red line separating the extracellular domain on the left and the intracellular domain on the right. The KR-rich segment is hydrophilic and therefore disordered. It is missing in the structure, so is the transmembrane domain. The 5-aa zinc-binding motif is highlighted within the green-line enclosure. **(C)** The tissue-specific expression data is extracted from The Human Protein Atlas (Uhlén et al., 2015).

ACE2 is a typical single-pass transmembrane receptor with a hydrophobic signal peptide of 17 aa and a single hydrophobic transmembrane domain (Figure 1A). ACE2 is a metallopeptidase with a 5-aa HEMGH zinc-binding motif (Figure 1A). Several proteases including TMPRSS2 (transmembrane serine protease 2), ADAM-17 (a disintegrin and metalloprotease 17, also known as TACE) and HAT (Human airway trypsin-like protease) can cleave ACE2 at the segment rich in lysine (K) and arginine (R) close to the transmembrane domain (Figure 1A) to shed enzymatically active soluble ACE2 (sACE2). This KR-rich segment is hydrophilic and consequently disordered, and is missing in the ACE2 structure (1R42, Figure 1B; Towler et al., 2004). What is particularly interesting is that such cleavage of ACE2 by proteases, especially by ADAM-17 (Haga et al., 2008, 2010; Scheller et al., 2011), is activated in SARS-CoV infection. One naturally would think that such cleavage might be a protective response by the host cells, i.e., if membrane-bound ACE2 mediates viral entry, then cleaving them off membrane would decrease infection. Surprisingly, the generation of sACE2 enhances infection (Haga et al., 2008, 2010). This shows the complexity in pathogen-host interactions that I will discuss in more detail later.

Ever since the first characterization of ACE2 (Donoghue et al., 2000; Tipnis et al., 2000), gene expression of ACE2 has been found high in kidney, heart, testis, colon and small intestine, but low in lungs (Hikmet et al., 2020; Li et al., 2020; Figure 1C). This low expression of ACE2 in lungs has motivated the search for alternative receptors and cofactors, until it was found that ACE2 is highly expressed in type II pneumocytes in lungs (Hamming et al., 2004; To et al., 2004; To and Lo, 2004; Mossel et al., 2008; Xu et al., 2020; Zhao et al., 2020), so the low expression in lungs is due to the mixture of these type II pneumocytes with other types of lung cells that express little ACE2. I should add that the “high” expression of ACE2 in type II pneumocytes is relative to other types of lung cells. The ACE2 expression in type II pneumocytes is still negligibly low relative to other cell types such as those in the digestive system (e.g., enterocytes) or connected to the digestive system (e.g., cholangiocytes), or cells in kidney (e.g., proximal tubular epithelial cells) or in testes (e.g., Sertoli cells), according to data in The Human Protein Atlas (Uhlén et al., 2015).

Interestingly, ACE2 was found to be expressed in oral tissues, especially in tongue (Xu et al., 2020), although the expression is generally low compared to that in the digestive tract. The expression of ACE2 in tongue indicates the potential of destruction of tongue cells upon COVID-19 infection. Whether this might be linked to the loss of taste, a common symptom of COVID-19 infection, has not been explored.

Other candidate receptors that have been used by various coronaviruses include amino peptidase N (APN) and dipeptidyl peptidase 4 (DPP4). However, only cells expressing ACE2 are susceptible to SARS-CoV-2 infection. The presence/absence of APN or DPP4 is irrelevant to SARS-CoV-2 infection (Zhou P. et al., 2020). While ACE2 binds to SARS-S and SARS-2S, DPP4 does not (Wang et al., 2020). In particular, ACE2 from mice which is substantially diverged from human ACE2 does not support viral entry (Zhou P. et al., 2020). However, transgenic mice expressing human ACE2 are vulnerable to SARS-CoV-2 and can develop COVID-19 symptoms (Bao et al., 2020), suggesting that ACE2 is a sufficient receptor for SARS-CoV-2 attachment and cell entry.

Further corroboration of the interaction between the viral RBD and the host ACE2 comes from microscopy methods and structural characterization. Microscopy methods such as confocal fluorescence microscopy can visualize the binding of coronavirus spike proteins to GFP-tagged ACE2 (Wang et al., 2020). Structural studies have characterized not only the structure of SARS-2S monomer and trimmer (Hoffmann et al., 2020; Walls et al., 2020; Wrapp et al., 2020; Yan et al., 2020), but also the SARS-2S trimer and the ACE2 in complex (Gui et al., 2017; Wang et al., 2020; Zhou T. et al., 2020; Xu et al., 2021). What remains to be elucidated is the mechanistic aspects of how the ACE2-binding triggers the transformation of the SARS-2S trimer from the prefusion state to the postfusion state.

These structural studies also provide a list of amino acids in physical contact with each other from the two interacting partners (Lu et al., 2015; Adhikari and Ching, 2020; Adhikari et al., 2020). The sharing of the interacting amino acids in ACE2 were subsequently used to predict what other mammalian species have an ACE2 that can serve as a host receptor for SARS-CoV-2 infection (Shi et al., 2020; Kruglikov et al., 2021; Wei et al., 2021), which I discuss later. Such information also facilitates the identification of key residues that contribute to the host tropism of SARS-CoV-2. For example, SARS-CoV-2 cannot infect mice because of differences in five key residues in ACE2 between mouse and human. Replacing these residues created a mouse model susceptible to SARS-CoV-2 infection (Adams et al., 2021).

Protein structures also shed light on interactions between the receptor and the viral spike proteins. For example, the structure (7KNB) of human ACE2 in complex with SARS-2S trimer (Zhou T. et al., 2020) shows four segments in ACE2 (19–39, 323–330, 352–357, 385–390) and two segments in SARS-2S (443–458, 472–506) to be in close physical proximity. The amino acids in the four ACE2 segments jointly have an isoelectric point (pI) of 4.38, and those in the two SARS-2S segments jointly have a pI of 9.40. Thus, at neutral pH, the former is negatively charged, but the latter is positively charged. The two therefore would have favorable electrostatic interactions facilitating their binding to each other. This result makes sense of a previous mutation experiment (Adams et al., 2021) to convert the mouse ACE2 that cannot bind to SARS-2S to one that can, based on the sequence difference between human and mouse ACE2. The two mutation constructs (hmACE2.3 and hmACE2.4) that introduced negatively charged amino acid residues present in human ACE2 into mouse ACE2 (N30D in hmACE2.3, and A329E in hmACE2.4) can functionally interact with SARS-2S just as well as human ACE2. The other two constructs (hmACE2.1 and hmACE2.2) introduced mutations to increase hydrophilicity (e.g., H353K in hmACE2.1 and N31K in hmACE2.2), which also improved the interaction of mouse ACE2 with SARS-2S, albeit to a smaller degree than hmACE2.3 and hmACE2.4.

It is important to keep in mind the difference between SARS-CoV and SARS-CoV-2 in their use of ACE2 for cell entry (Xia, 2021). First, there are two documented alternative pathways of cell entry for coronaviruses after receptor binding: (1) cell entry by membrane fusion when the spike trimer is cleaved at the polybasic furin site, and (2) cell entry by clathrin-mediated endocytosis (Inoue et al., 2007) and the endosome-cathepsin pathway (Matsuyama et al., 2005, 2010). SARS-CoV-2, with the cleaved furin site, uses mainly pathway 1, whereas SARS-CoV uses pathway 2. The inhibition of the clathrin-mediated endocytosis dramatically reduces cell entry by SARS-CoV (Inoue et al., 2007). SARS-CoV cannot use pathway 1 because of the lack of the polybasic furin site. However, a polybasic furin site experimentally introduced into SARS-CoV at the same location as in SARS-CoV-2 created a much more infectious SARS-CoV (Belouzard et al., 2009) with syncytium formation characteristic of SARS-CoV-2 infection. Similarly, SARS-CoV-2 lacking the polybasic furin site are less infective with little syncytium formation (Peacock et al., 2021). Second, SARS-CoV-2 infection is frequently associated with syncytia formation (Daly et al., 2020; Hoffmann et al., 2020; Li et al., 2021) which is rarely reported with SARS-CoV infection. This syncytia formation implies that, once SARS-CoV-2 has entered a cell, it can infect neighboring cells without using ACE2. Thus, high ACE2 abundance in young people than old people (Plaas et al., 2021; Bastolla et al., 2022) renders them more susceptible to SARS-CoV than old people. However, the reduced dependence of SARS-CoV-2 on membrane ACE2 allows SARS-CoV-2 to infect old people who are immunologically weak (Montecino-Rodriguez et al., 2013), even though they do not express a high level of ACE2.

### 2.2. Other candidate receptors and cofactors

The identification of ACE2 as the host receptor does not imply that it is the only host receptor. Several viruses are known to use multiple receptors and co-factors. For example, Dengue virus uses both human mannose-binding receptor (MR) and DC-SIGN on macrophages as primary receptors (Lo et al., 2016), and HIV-1 uses both CD4 as a primary receptor and a cellular coreceptor (Wilen et al., 2012). Are there other receptors or cofactors that facilitate SARS-CoV-2 attachment and cell entry? Existing evidence points to a hypothesis that has not yet been fully explored. SARS-CoV-2 can bind to both membrane-bound ACE2 or soluble sACE2 which could then bind to membrane proteins such as neuropilin-1 (Cantuti-Castelvetri et al., 2020; Daly et al., 2020), integrins (Nader et al., 2021; Nader and Kerrigan, 2022), or other membrane proteins to anchor SARS-CoV-2 to host cell membrane.

If SARS-S and SARS-2S use ACE2 as the only host receptor to mediate attachment and cell entry, then SARS-CoV and SARS-CoV-2 should infect the same tissue. However, the two viral lineages differ in their tissue tropism, with SARS-CoV more likely infecting lower respiratory tract than SARS-CoV-2. Two mutations in SARS-2S relative to SARS-S have been hypothesized to contribute to differences in cell tropism between SARS-CoV and SARS-CoV-2, and to involve alternative receptors or cofactors.

#### 2.2.1. Polybasic furin site and NRP1 (neuropilin-1)

One conspicuous difference between SARS-S and SARS-2S is the presence of a polybasic furin site, RRAR^S, in the latter but not in the former (Andersen et al., 2020; Coutard et al., 2020; Hoffmann et al., 2020). Two lines of evidence suggests that this furin site is responsible for the difference in tissue tropism between SARS-CoV and SARS-CoV-2. First, a previous study demonstrated that inserting a polybasic furin site at the S1 and S2 boundary in SARS-S resulted in dramatic changes in cell tropism (Belouzard et al., 2009). Second, the spike protein trimer in SARS-CoV-2 virions is already cleaved at this furin site to prime the fusion between the viral and host membranes (Hoffmann et al., 2020; Xia, 2021), and the C-terminus of the cleaved S1 is accessible for interacting with other proteins (Walls et al., 2020; Wrapp et al., 2020). One may therefore infer that host membrane proteins with structural affinity to the cleaved end of SARS-CoV-2 could serve either as an alternative host receptor or an enhancer of viral infection.

**Figure 2:**
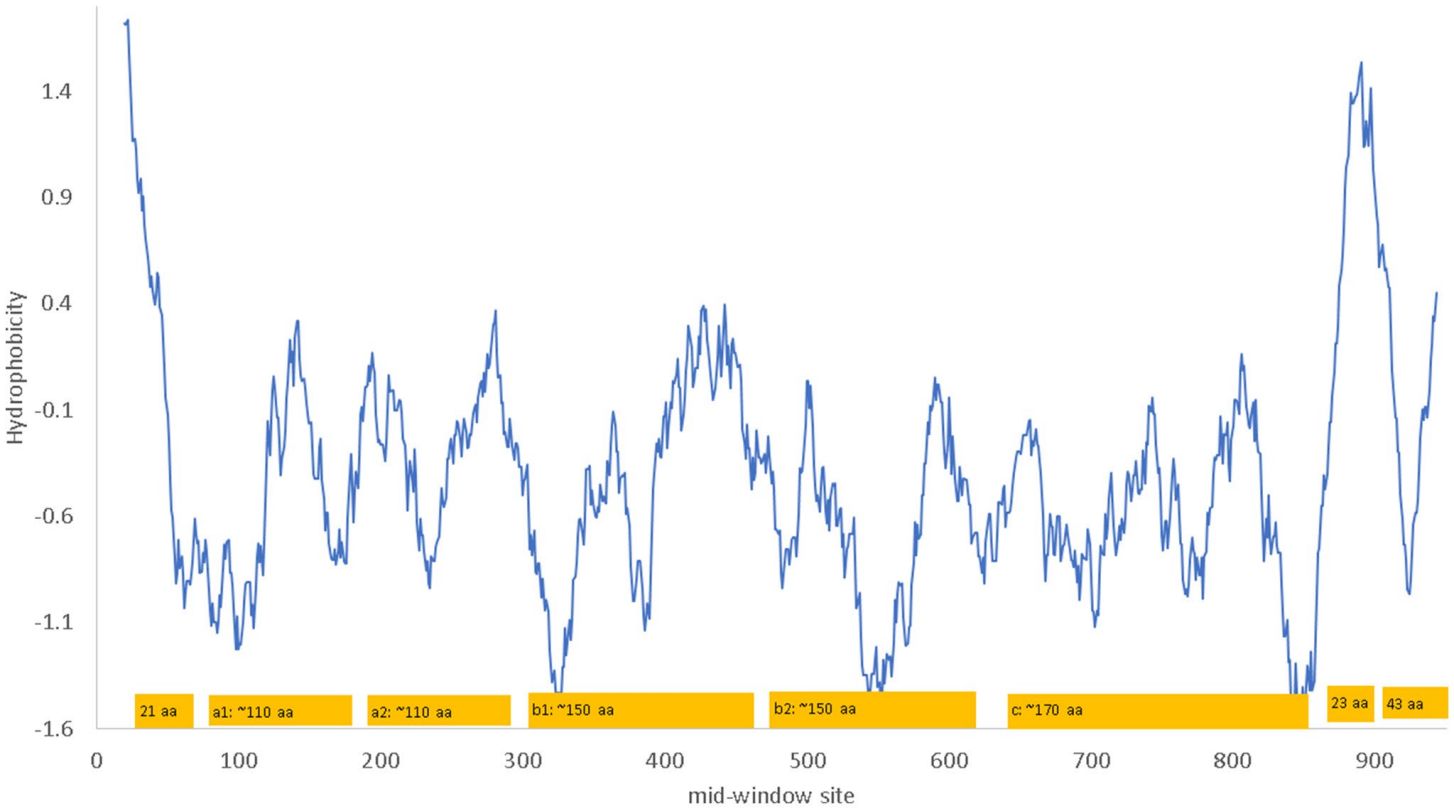
Hydrophobicity plot and domain structure of human neuropilin-1 (NRP1) along a sliding window of 40 amino acids (aa). The b1-b2 domains bind to the furin-cleaved C-terminus of the S1 subunit of SARS-CoV-2. The signal peptide includes the first 21 aa. The 23 aa near the C-terminus of NRP1 constitute the single-pass transmembrane domain that divides the NRP1 protein into the long extracellular domain and the short 43-aa cytoplasmic domain. The hydrophobicity plot was generated from DAMBE (Xia, 2018b) based on hydrophobicity values in Kyte and Doolittle (1982). The domains are not drawn exactly to scale. The numbering of amino acids on the horizontal axis follows the neuropilin-1 isoform X1 annotated on human chromosome 10 (NC_000010).

NRP1 is a single-pass membrane protein which is obvious from a hydrophobicity plot (Figure 2) as it has just a single hydrophobic transmembrane domain. The hydrophobic stretch of 21 amino acids at the N-terminus is the signal peptide (Figure 2). The a1 and a2 domain are also known as the CUB domains. The b1-b2 domains bind to the furin-cleaved C-terminus of the S1 subunit of SARS-CoV-2 (Daly et al., 2020). The 23-aa segment near the C-terminus of NRP1 is the single-pass transmembrane domain that divides the NRP1 protein into the long extracellular domain and the short 43-aa cytoplasmic domain.

NRP1 is a receptor for other glycoproteins such as VEGF-A and SEMA3A (Plein et al., 2014). Its b1-b2 domain binds specifically to furin-cleaved substrates that has an R/KXXR/K motif at the C terminus where X is any amino acid (Teesalu et al., 2009; Plein et al., 2014). Experimentally determined NRP1 structure shows the negatively charged D320 in NRP-1 interacting electrostatically with the positively charged R/K residue at the C-terminus of the ligand (Guo and Vander Kooi, 2015), and a ligand with the terminal R/K removed may serve as an NRP1 inhibitor. The furin-cleaved S1 subunit of SARS-2S, with the C-terminal RRAR conforming to the R/KXXR/K motif, binds directly to NRP1 (Daly et al., 2020). The following three experimental studies demonstrated NRP1 to be a cofactor that enhances ACE2-mediated viral attachment and cell entry, although it does not serve a sufficient host receptor for SARS-CoV-2 independent of ACE2 (Cantuti-Castelvetri et al., 2020; Daly et al., 2020). First, blocking the binding between the b1-b2 domain and the C-terminus of the viral S1 subunit significantly reduces viral internalization (Cantuti-Castelvetri et al., 2020). Second, removing the RRAR at the C-terminus of the S1 subunit of SARS-2S decreases the binding of S1 to NRP1, and knocking out NRP1 decreases SARS-CoV-2 infection of Hela cells expressing ACE2 (Daly et al., 2020). Third, x-ray crystallography and biochemical approaches revealed that NRP1 enhances internalization of SARS-CoV-2 and syncytia formation (Daly et al., 2020) that has been observed previously to enhance SARS-CoV-2 propagation from cell to cell (Hoffmann et al., 2020).

**Figure 3:**
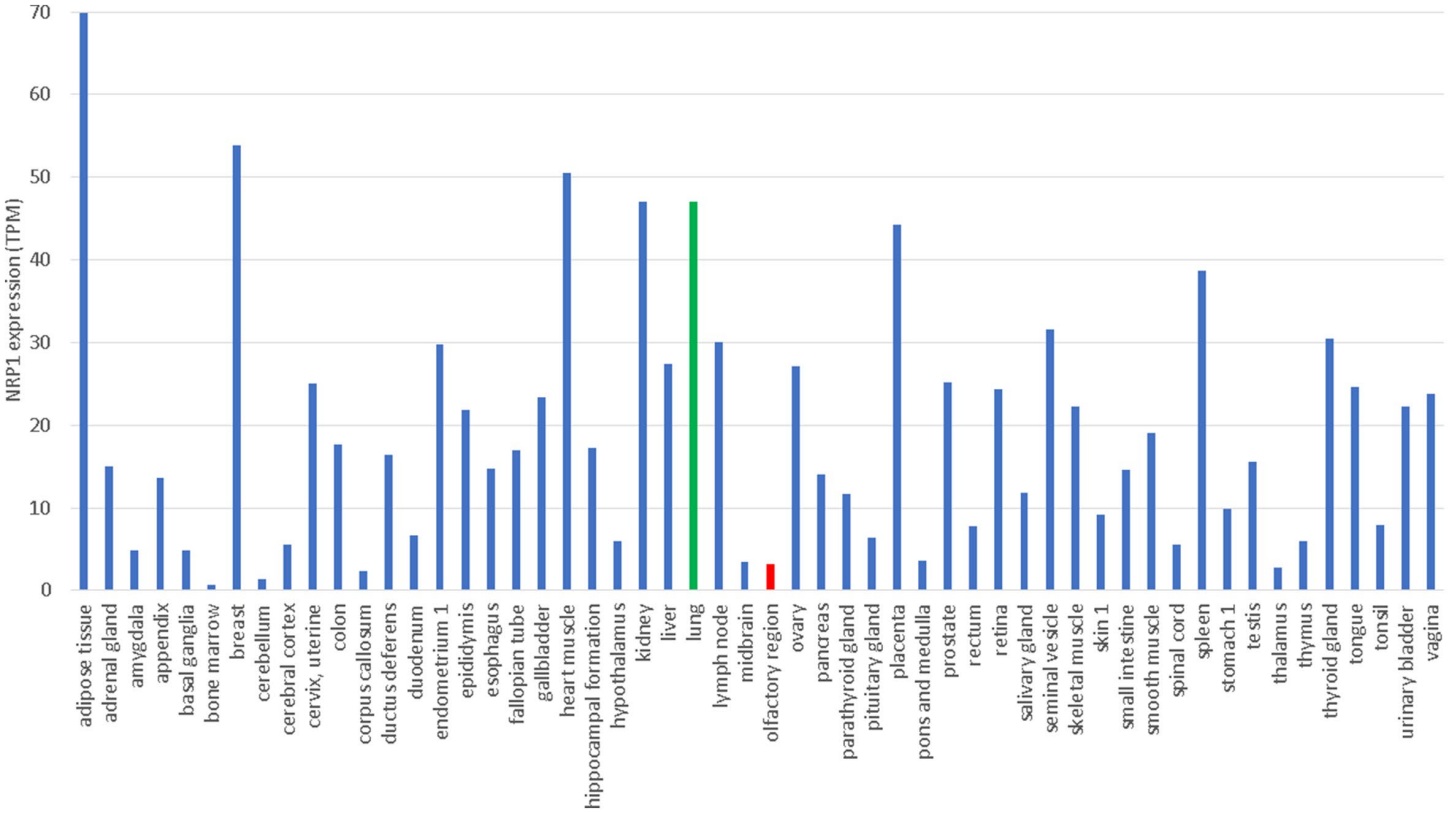
Tissue-specific expression of *NRP1* from The Human Protein Atlas (Uhlén et al., 2015), in unit of TPM (transcripts per million). Gene expression in lung and olfactory regions is colored green and red, respectively.

NRP1 is highly expressed in the olfactory epithelium, and the SARS-CoV-2 infection appears to be correlated with NRP1 expression (Cantuti-Castelvetri et al., 2020). This could explain why SARS-CoV-2 infects predominantly the upper respiratory tracts, in contrast to SARS-CoV that infects lower respiratory tracts and lungs. However, NRP1 is also abundantly expressed in almost all pulmonary tissues (Cantuti-Castelvetri et al., 2020) including lungs (Figure 3), so more studies are needed to check if patients with SARS-CoV-2 infection of lungs also have higher expression of NRP1 in lungs than those without lung infections.

#### 2.2.2. The K403R mutation in SARS-2S, the resulting RGD motif and integrins

**Figure 4:**
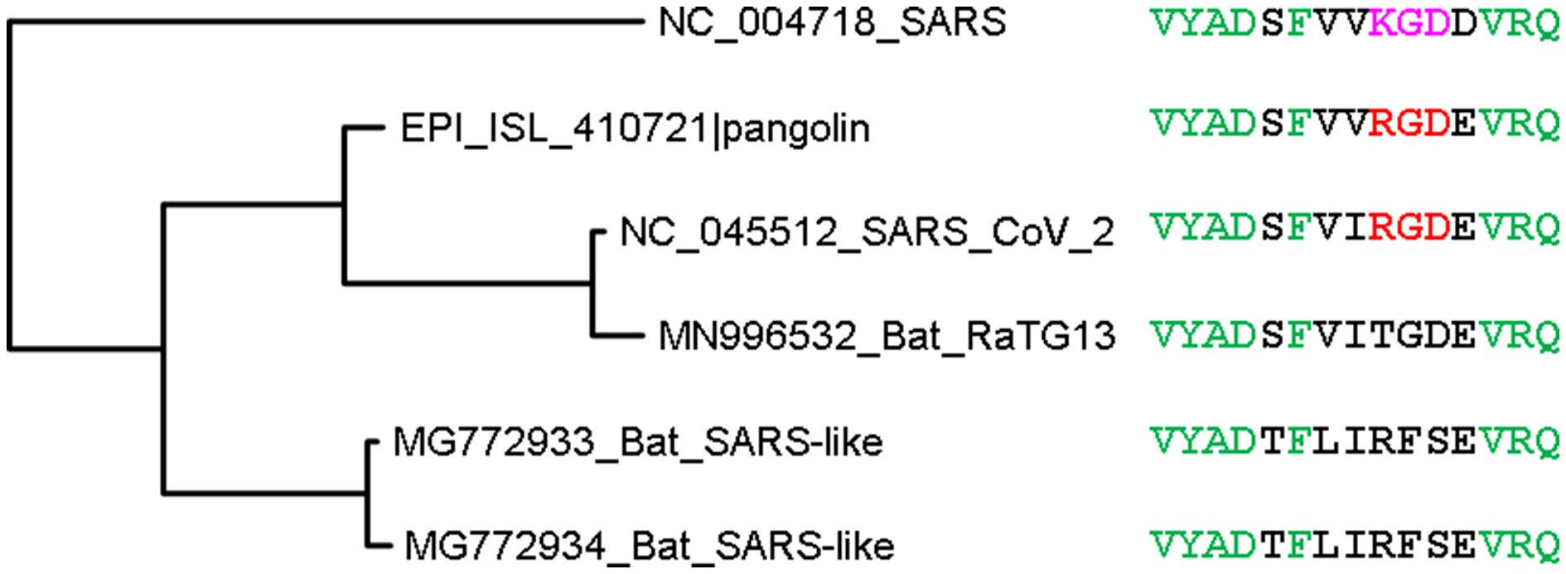
Phylogenetic tree of spike protein sequences from SARS-CoV-2 and close relatives. OTU names are in the form of accession (GenBank or GISAID) followed by viral strain designation. The protein sequences were aligned by MAFFT (Katoh and Toh, 2008) with the accurate but slow L-INS-i option. The unrooted phylogenetic tree was reconstructed with PhyML (Guindon and Gascuel, 2003), with the empirical LG substitution matrix and optimization of topology, branch lengths and rates. Identical sites are colored green. The RGD motif, colored red, is shared between SARS-CoV-2 and a close relative isolated from pangolin, and differ from the KGD motif in SARS-CoV by a conservative K403R mutation. The RGD motif is the binding target of the major endothelial cell integrin, αVβ3.

Another mutation in SARS-2S relative to SARS-S is K403R (Figure 4) which creates an RGD motif known to be a general integrin-binding motif (Takada et al., 2007). This motif is shared between SARS-CoV-2 and its close relatives isolated from pangolins. The homologous motif in SARS-CoV is KGD (Figure 4). Thus, both SARS-2S and the spike protein from pangolin-isolated SARSr (where r stands for coronaviruses closely related to SARS) are expected to bind to integrins, especially the major endothelial cell integrin, αVβ3 (Nader et al., 2021; Nader and Kerrigan, 2022).

**Figure 5:**
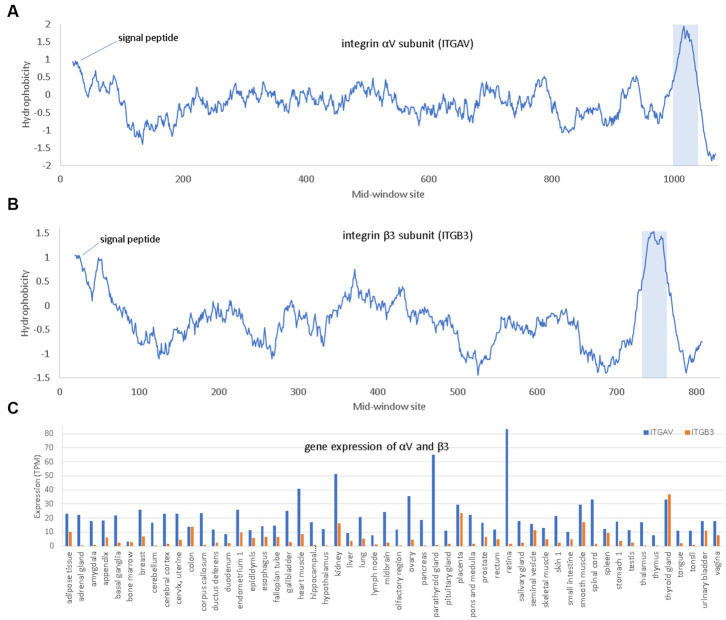
Hydrophobicity plot and gene expression for the two subunits (αV and β3) of the major endothelial integrin αVβ3. **(A,B)** Hydrophobicity plot for αV and β3, respectively, with gene names *ITGAV* and *ITGB3*, respectively, along a 40-aa window. **(C)** Gene expression for αV and β3 in different tissues extracted from The Human Protein Atlas (Uhlén et al., 2015), in unit of TPM (transcripts per million). At the N-terminus is the hydrophobic signal peptide. The hydrophobic transmembrane domain is shaded.

Integrins are membrane receptors existing as αβ heterodimers (Figure 5). Like NRP1, both α and β subunit of integrins are single-pass membrane proteins with a single transmembrane domain, illustrated with αV and β3 subunits (Figure 5). Human genomes encode at least 18 α subunits and eight β subunit. The RGD-recognizing integrins include α5β1, αVβ1, αVβ3, αVβ5, αVβ6, αVβ8, and αIIbβ3 (Takada et al., 2007). Most integrins are localized to specific tissues but the major endothelial cell integrin, αVβ3, is widely distributed in endothelium (Takada et al., 2007). The αV subunit has multiple β partners to form heterodimers, but β3 subunit form heterodimers mainly with αV. For this reason, αV can be highly expressed in tissues without β3 because αV has other β partners, but β3 is expressed mainly in tissues with αV (Figure 5).

Three interesting findings were derived from *in-silico* molecular simulation (Nader et al., 2021). Firstly, both RGD in SARS-2S and KGD in SARS-S (Figure 5) are located in a long flexible loop (PDB ID 6M0J for SARS-2S and 5XLR for SARS-S) free to interact with other proteins. However, R is larger in volume than K (124 vs. 119), so RGD in SARS-2S is more solvent exposed than KGD in SARS-S. Secondly, the RGD motif fits nicely into the ligand-binding pocket of the host αVβ3. Thirdly, the RGD motif is located about 32 aa upstream of the receptor-binding domain (RBD) for ACE2, and the interaction between the RGD motif in SARS-2S and the host αVβ3 appears physically independent of the interaction between the viral RBD and the host ACE2. Therefore, the host αVβ3 could serve as an additional receptor for SARS-2S independent of ACE2.

Three lines of experimental evidence support the hypothesis that αVβ3 may serve as an alternative receptor (Nader et al., 2021). First, SARS-2S bounds strongly to αVβ3 *in vitro.* Second, SARS-CoV-2 binds strongly to endothelial cells (which could be due to binding of SARS-2S to ACE2, αVβ3, or any other potential receptors). Third, the binding between SARS-CoV-2 and endothelial cells can be inhibited by Cilengitide (a specific αVβ3 antagonist), which suggests that the binding between SARS-CoV-2 and endothelial cells is mediated by αVβ3. However, the consequence of the binding between SARS-CoV-2 and endothelial cells is not clear. The binding could lead to cell entry of SARS-CoV-2, which would qualify αVβ3 as an alternative receptor. The binding could also interfere with the normal function of αVβ3 which participates in many cellular processes including angiogenesis, cell adhesion and migration, and signaling (Takada et al., 2007), leading to loss of vascular barrier integrity and consequently enhance SARS-CoV-2 infection and increase the severity of COVID-19 (Nader and Kerrigan, 2022).

One may argue that the expression of αVβ3 mainly in endothelial cells would limit its availability for SARS-CoV-2 infection, i.e., SARS-CoV-2 would need to first infect epithelial cells and then traverse to endothelial cells to access αVβ3 as a host receptor. In contrast, ACE2 is expressed in both epithelial and endothelial cells (Hamming et al., 2004). However, the epithelial cells and the endothelial cells are separated by only a very thin basement membrane in lungs. There are also integrins that are expressed in epithelial cells. For example, α5β1, which also binds to the RGD motif, is expressed in a variety of cells including epithelial cells in digestive tract (Sheppard, 1996). Human α5β1 was also implicated in SARS-CoV-2 infection (Robles et al., 2022), and inhibition of human α5β1 by its inhibitor ATN-161 has been shown to reduce viral load in k18-hACE2 transgenic mice infected with SARS-CoV-2 (Amruta et al., 2021; Beddingfield et al., 2021).

How important the RGD motif is in binding to αVβ3 or α5β1 in terms of sequence context could be investigated by either mutating the KGD motif in SARS-S to RGD or investigating the binding properties of the RGD-containing spike protein of the pangolin-derived SARSr. The latter can be done by *in-silico* protein docking and dynamic modelling. Whether the RGD motif binds to integrins as hypothesized above remains controversial (Zech et al., 2021; Othman et al., 2022). Structural modelling of molecular dynamics suggests that the RGD motif does not bind to integrin (Othman et al., 2022).

**Figure 6:**
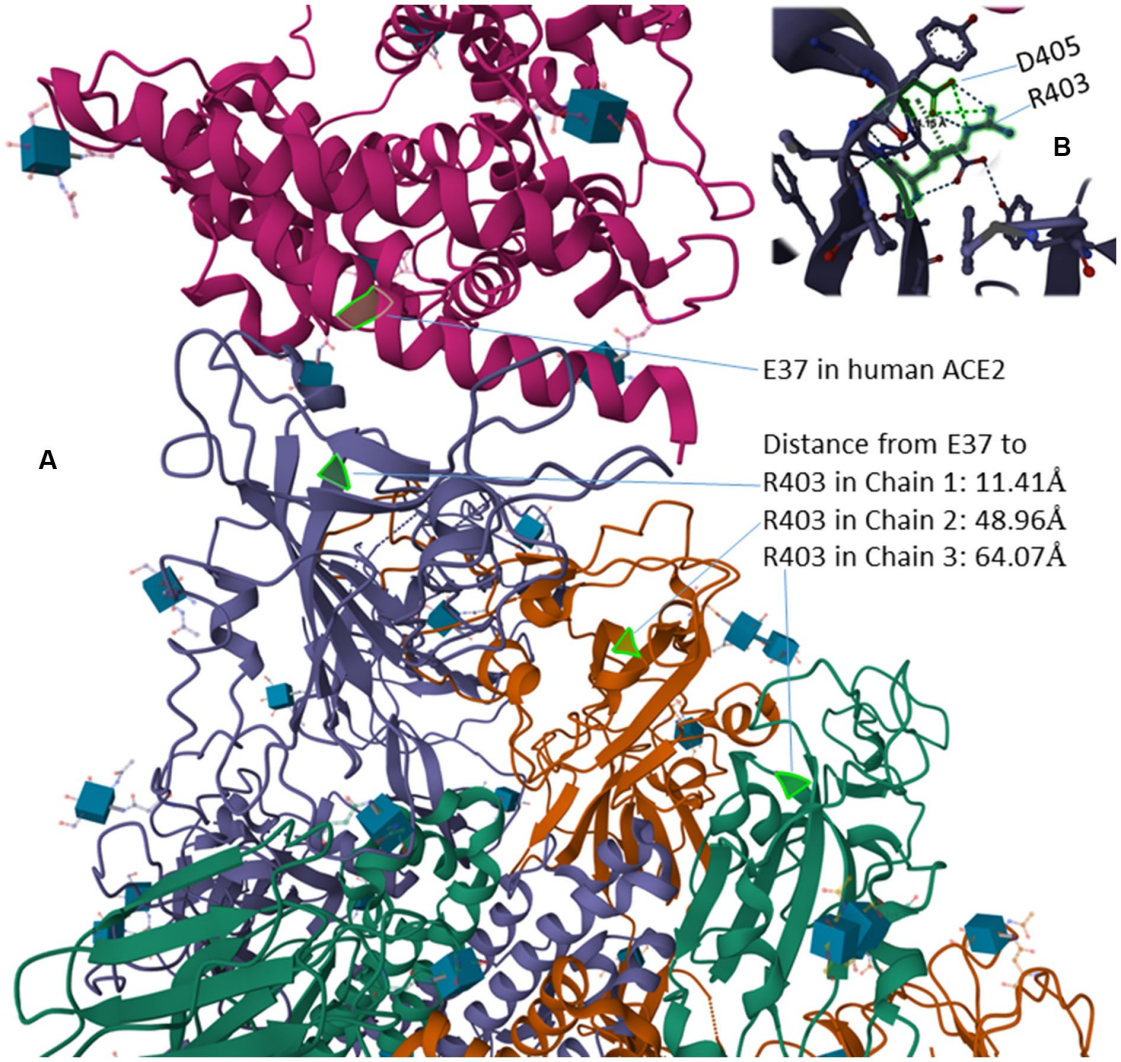
Protein structure of human ACE2 in complex with the SARS-2S trimer (PDB accession 7KNB) (Zhou T. et al., 2020). **(A)** The distance from the negatively charged E37 in ACE2 to the three positively charged R403 residues, one in in each of the three SARS-2S monomers. **(B)** R403 and D405 within SARS-2S, which are 4.15 Å apart, interact with each other electrostatically.

The R residue in the RGD motif is experimentally shown to enhance binding of the virus to human cells and subsequent viral entry into the cell (Zech et al., 2021). In the bat-derived virus RaTG31, the closest relative of SARS-CoV-2, the site homologous to R403 is T403 (Figure 4). The spike protein of RaTG13 is weak in binding to human ACE2 relative to SARS-2S (Li et al., 2021). Replacing T403 in RaTG13 by R403 enhances viral receptor binding and viral entry into human cells (Zech et al., 2021). Similarly, replacing R403 in SARS-2S by T403 reduces the viral binding and cell entry (Zech et al., 2021). However, R403 was interpreted to enhance the binding between SARS-2S and ACE2, especially between positively charged R403 in SARS-2S and negatively charged E37 in ACE2, but not between SARS-2S and integrin (Zech et al., 2021). This interpretation, based only on structural modelling (Zech et al., 2021), is probably tenuous. The experimentally determined structure (7KNB) of human ACE2 in complex with the SARS-2S trimer (Zhou T. et al., 2020) shows that R403 in SARS-2S and E37 in ACE2 are not close physically (Figure 6A). Of the three R403 residues, one in each of the SARS-2S monomers, the closest distance between E37 and R403 is 11.41 Å apart (Figure 6A). I should mention that there are many different formulations of inter-residue distances. The first (and the simplest) is the distance between the alpha-carbon in one residue and the alpha-carbon in the other residue. This tends to be the most stable across different experimentally determined structures, and is the distance in Figure 6A. The second is to first calculate the centroid for each amino acid, and then calculate the distance between the two centroids. The third is to compute the centroid of the interacting functional groups, e.g., the amino group in Lys and the carboxyl group in glutamate, and then compute the distance between the two centroids.

Instead of interaction between R403 and E37, the structure (7KNB) shows electrostatic interaction between positively charged R403 side chain and the negatively charged D405 side chain (Figure 6B), the two being 4.15 Å apart. The force of attraction between oppositely charged side chains decreases with $d^{2}$ (where d is the distance between the interacting partners), so the electrostatic interaction between R403 and D405 within SARS-2S should be much stronger than that between R403 in SARS-2S and E37 in human ACE2. The structural relationship among residues appears consistent across different structural experiments. For example, when human ACE2 is in complex with a SARS-2S monomer instead of a trimer, the distance between R403 in SARS-2S and E37 in ACE2 is 11.15 Å (negligibly smaller than the previous 11.41 Å). Thus, the structure does not suggest a strong interaction between the RGD motif and ACE2, so the RGD motif is free to interact with others, including integrins.

However, there could be an indirect interaction between SARS-2S and integrin through soluble ACE2 (sACE2) as follows. Membrane proteins ADAM-17 and TMPRSS2 cleave the extracellular domain of ACE2 generating sACE2 (Donoghue et al., 2000; Kuba et al., 2010; Scheller et al., 2011; Heurich et al., 2014). sACE2, which features its own RGD motif at sites 204–206, can bind to integrins either in an RGD-dependent or an RGD-independent manner (Clarke et al., 2012). SARS-2S could first bind to sACE2 and then brought close to integrin through sACE2-integrin binding. This is consistent with the observation that shedding of ACE2 results in increased uptake of SARS-CoV virions into host cells (Haga et al., 2008, 2010; Heurich et al., 2014).

There has been insufficient exploration of the functional consequence of the K403R change. Lysine acetylation occurs in both nucleus and cytoplasm (Sadoul et al., 2011; Mu et al., 2020) and removes the positive charge of the lysine residue. Because the lysine in the KGD motif in SARS-S is located in a long flexible loop, it could be acetylated and lose its potential to interact electrostatically with a negative amino acid residue. In contrast, R403 will always be positively charged under normal cellular or tissue pH. It is consequently important to know if K403 in SARS-S is acetylated during virion assembly.

#### 2.2.3. Other candidate receptors and cofactors requiring further empirical confirmation

**Figure 7:**
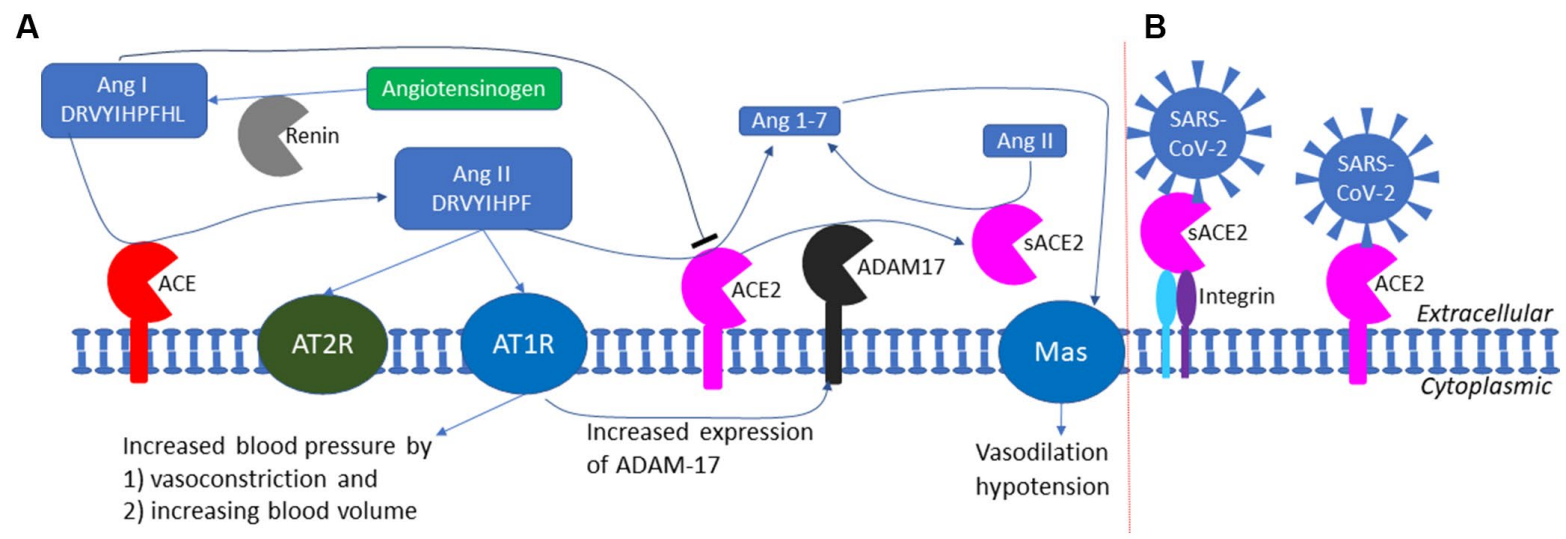
The RAS (renin-angiotensin system) and the exploitation of ACE2 by SARS-CoV-2 as a cell receptor. **(A)** The RAS system in maintaining blood pressure homeostasis through negative feedback. Low blood pressure triggers the release of renin which cleaves angiotensinogen (colored green) to produce Ang I; ACE cleaves Ang I to generate Ang II which binds to AT1R receptor to increase blood pressure; ACE2 degrade Ang II to prevent prolonged hypertension. ADAM17 cleaves the extracellular domain of ACE2 to generate soluble ACE2 (sACE2) which is also enzymatically active in degrading Ang II. **(B)** SARS-CoV-2 could anchor itself to the cell membrane by binding either to ACE2 or to sACE2 through other membrane proteins such as integrin (not to scale).

It has also been suggested that kidney injury molecule-1 (KIM1) may serve as an alternative host receptor for SARS-S and SARS-2S (Yang et al., 2021). However, the evidence is not strong, and the argument that ACE2 alone cannot explain the kidney impairment associated with COVID-19 infection is weak. ACE2 is more highly expressed in kidney than in lung based on tissue-specific expression of protein-coding genes (Fagerberg et al., 2014; Uhlén et al., 2015), as well as on ACE2 activity assays in diabetic mice (Wysocki et al., 2006), which seems sufficient to explain the susceptibility of kidneys to COVID-19 infection without any need to invoke alternative receptors. Kidney impairment associated with COVID-19 can be explained by the impairment of ACE2 function in degrading Ang II (Figure 7A). ACE2 protects kidneys from unchecked RAS responses including hypertension, inflammation and tissue damage (Kuba et al., 2010; Soler et al., 2013). COVID-19 infection in kidney destroys kidney cells expressing ACE2 and exposes the kidneys to unchecked RAS responses causing kidney impairment.

Two other membrane proteins, AXL and CD147, may deserve attention. AXL is a putative cell receptor for Zika virus (Nowakowski et al., 2016), and implicated in mediating cell entry via the endosome pathway by SRAR-CoV-2 (Bohan et al., 2021). CD147 is a membrane glycoprotein known to be involved in infection by eukaryotic, prokaryotic and viral pathogens (Fenizia et al., 2021), and may also bind to SARS-2S and mediate the cell entry of SARS-CoV-2 through endocytosis (Brodowski et al., 2022; Cavezzi et al., 2022; Kalejaiye et al., 2022), although infection mediated by CD147 is most likely secondary because CD147 is highly expressed in neural tissues but not in respiratory tract (Qiao et al., 2020). Both genes appear weakly expressed in lungs based on tissue-specific gene expression data in The Human Protein Atlas (Uhlén et al., 2015). However, as I mentioned before, ACE2 is highly expressed in type II pneumocytes in lungs (Hamming et al., 2004; To et al., 2004; To and Lo, 2004; Mossel et al., 2008; Xu et al., 2020; Zhao et al., 2020), so the low tissue-specific expression of these two genes may not exclude the possibility of high expression in certain types of cells.

Another cofactor proposed to bind SARS-2S and facilitate SARS-CoV-2 cell entry is sialic acid-containing glycolipids (Nguyen et al., 2022). Depletion of these glycolipids decreases SARS-CoV-2 infection. However, the depletion of these glycolipids could have multiple consequences. It may impair membrane integrity and render epithelial cells more exposed. For example, mucins are important membrane component, and the loss of mucins enhances SARS-CoV-2 infection (Biering et al., 2022). Also, loss of membrane integrity may cause shedding of ACE2 and candidate cofactors such as NRP1 and consequently generate outcomes that are difficult to interpret.

The hypothesis of sialic acid-containing glycolipids or sialylated glycans as a receptor for SARS-2S is vague because many membrane proteins are sialylated glycoproteins, including ACE2 and CD147. However, ACE2 glycan processing has little effect on SARS-CoV-2 recognition (Allen et al., 2021). One may therefore infer that it is features other than sialylated glycans that is important in mediating SARS-CoV-2 infection.

## 3. Host receptors and cofactors as drug targets?

ACE2 has been studied as a drug target ever since it was identified as the host receptor for SARS-CoV. The rationale seems straightforward. Given that ACE2 is a gate to let SARS-CoV-2 into the cell, the gate should be blocked. Two questions need to be answered. First, does ACE2 abundance really increase the risk to COVID-19 (where “risk” is a term combining the vulnerability to COVID-19 and the severity of COVI-19 symptoms)? Second, how should ACE2 be targeted to reduce the risk to COVID-19 without interfering with the essential function of ACE2?

### 3.1. Does ACE2 abundance increase with the risk to COVID-19?

There are no direct experiments on ACE2 abundance and the risk to COVID-19. Consequently, an indirect approach has been used to address the question. COVID-19 symptoms are more severe in old-age group (OG) than the young or middle age group (YG). ACE2 abundance was obtained from different age groups to establish the relationship between ACE2 abundance and age. If OG expresses more ACE2 than YG, then ACE2 abundance is a likely contributor to the severity of COVID-19 in OG.

In a well-planned comparative study among groups of different ages and ACE2 expression in a hospital cohort (Plaas et al., 2021), ACE2 expression is higher in YG than in OG. Similarly, careful and structured meta-analysis also supports higher ACE2 in YG than in OG (Bastolla et al., 2022). However, large-scale compilation of data sometimes leads to contradictory results (Zheng, 2022). Some of the discrepancy could be explained by pooling unbalanced data. For example, if ACE2 expression levels in YG and OG in region 1 are $ACE2_{YG.r1}=20$ and $ACE2_{OG.r1}=10$ , respectively, but $ACE2_{YG.r2}=40$ and $ACE2_{OG.r2}=30$ in region 2 (where the subscript r stands for region). If sample size is $n_{YG.r1}=200$ and $n_{OG.r1}=20$ , but $n_{YG.r2}=5$ 0 and $n_{OG.r2}=500$ , then the weighted mean of ACE2 expression for YG and OG, pooled over the two regions, would become


$$\begin{array}{l} Mean_{YG}=\frac{ACE2_{YG.r1}\times n_{YG.r1}+ACE2_{YG.r2}\times n_{YG.r2}}{n_{YG.r1}+n_{YG.r2}}\\ \;\;\;\;\;\;\;\;\;\;\;\;\;\;\;\;\;\;\;\;\;\;=\frac{20\times 200+40\times 50}{200+50}=24 \end{array}$$

$$\begin{array}{l} Mean_{OG}=\frac{ACE2_{OG.r1}\times n_{OG.r1}+ACE2_{OG.r2}\times n_{OG.r2}}{n_{OG.r1}+n_{OG.r2}}\\ \;\;\;\;\;\;\;\;\;\;\;\;\;\;\;\;\;\;\;\;\;\;\;=\frac{10\times 20+30\times 500}{20+500}\approx 29.23 \end{array}$$

These two mean values would mislead us to conclude that ACE2 expression is higher in OG than in YG. This Simpson paradox, typically illustrated with the data from surgery on kidney stone data (Xia, 2018a), is often forgotten in large-scale data compilations.

The observation that OG has lower ACE2 abundance than YG (Plaas et al., 2021; Bastolla et al., 2022) seems incompatible with the observation that OG suffers more from COVID-19 than YG. There are two explanations. First, although ACE2 is higher in YG than in OG, the level of ACE2 in OG is still sufficient for initiating SARS-CoV-2 infection. Second, as I mentioned before, SARS-CoV-2 infection is associated with syncytia formation. This means that, once SARS-CoV-2 infected a cell, the spread of SARS-CoV-2 from this infected cell to neighboring uninfected cell may not need ACE2. In contrast to SARS-CoV-2, SARS-CoV infection does not form syncytia, so infection of new cells requires ACE2. Because ACE2 is more abundant in YG than in OG, people in YG tend to have higher risk to SARS-CoV than those in OG, which is consistent with SARS epidemiological data. I should emphasize that previous studies quantifying ACE2 expression does not take into consideration the sACE2 (the soluble portion of ACE2), so one should be cautious in interpreting ACE2 abundance and COVID-19 risk in different age groups.

### 3.2. Targeting ACE2 to reduce the COVID-19 risk without impacting ACE2 function

ACE2 has multiple functions (Fyhrquist and Saijonmaa, 2008; Kuba et al., 2010), but its most well-documented function is to buffer the RAS (renin-angiotensin system) effect for blood pressure homeostasis (Figure 7A). Human liver produces the 485-aa angiotensinogen which, after cleaving the 33-aa N-terminal signal peptide (Kumar et al., 2011), is released as the 452-aa mature circulating angiotensinogen (Figure 7A). When blood pressure falls, renin released from kidney cells converts angiotensinogen to Ang I, with cleavage between 10 L and 11 V (Yan et al., 2019). Ang I is in turn converted by ACE, a peptidyl dipeptidase, to Ang II (Figure 7A). Ang II interacts with the two receptors, but mainly through receptor AT1R (Figure 7A), to increase the blood pressure by (1) increasing the blood volume and (2) shrinking the blood vessel (vasoconstriction). This RAS function, if unchecked, would lead to hypertension, inflammation, tissue damage, heart failure, and other cardiovascular abnormalities (Kuba et al., 2010). Carboxypeptidase ACE2 takes short oligopeptides such as peptide hormones and cleaves efficiently at the Pro^X junction (where X is a hydrophobic amino acid at the C-terminus) (Donoghue et al., 2000; Tipnis et al., 2000; Dales et al., 2002). This reduction in Ang II, together with the binding of the resulting Ang1-7 to MAS receptors (Figure 7A), buffers the RAS effect to maintain blood pressure homeostasis. People with low levels of ACE2 tend to have high level of Ang II and hypertension, and need to be treated with ACE inhibitors so that Ang I is not converted to Ang II (Imai et al., 2005; Kuba et al., 2010). Alternatively, one may use drugs such as griseofulvin (a known vasodilator) which decreases blood pressure (Rubin, 1963; Aldinger, 1968). A recent study suggests that the griseofulvin effect may be mediated by its binding to ACE2 (Aris et al., 2022), i.e., griseofulvin may be an ACE2 enhancer.

Oligopeptides with His^X at the C-terminus can also serve as substrates for ACE2, although the cleavage is not as efficient as Pro^X (Dales et al., 2002). ACE2 can therefore cleave the terminal leucine in Ang I (Figure 7A). However, Ang I also inhibits ACE2 activity (Dales et al., 2002; Figure 7A), which is essential for the accumulation of Ang II. High levels of Ang I indicates weak activity of ACE and weak RAS effect, so ACE2 should be at low activity as well. When Ang I is converted to Ang II, the inhibitory effect of Ang I on ACE2 is removed, and the active ACE2 clears Ang II to prevent hypertension.

Ignoring the sACE2 activity in degrading Ang II may lead to misunderstanding of the negative feedback regulation of the RAS system. For example, an increase in Ang II level was associated with a decrease in myocardial ACE2 protein level (Patel et al., 2014). If one takes the decreased myocardial ACE2 protein level as decreased ACE2 activity, then one would conclude that an increase in Ang II, instead of increasing the ACE2 activity to degrade Ang II, actually decreases the ACE2 activity. This would imply a prolonged high concentration of Ang II because such a high Ang II concentration would seem to decrease ACE2 that degrade Ang II, so an increase in Ang II would lead to further increase in Ang II. However, the observed decrease in myocardial ACE2 may not imply decreased ACE2 activity because such decrease in myocardial ACE2 may be associated with an increase in sACE2 (Figure 7A). Because sACE2 is also enzymatically active in degrading Ang II (Kuba et al., 2010; Patel et al., 2014), the total ACE2 activity may not be decreased even though the myocardial ACE2 level is decreased. One needs to measure total ACE2 activity, including both the membrane-bound ACE2 and sACE2, in converting Ang II to Ang1-7 (Figure 7A).

Given the essential function of ACE2, simply downregulating ACE2 expression to reduce SARS-CoV-2 infection (Brevini et al., 2023) may incur the side effect of insufficient ACE2 activity. However, low ACE2 activity could be compensated by inhibitors of ACE such as MLN4760 (Dales et al., 2002) or angiotensin-receptor blocker (ARB) (Kuba et al., 2010; Bosso et al., 2020) or vasodilators such as griseofulvin (Aris et al., 2022). The binding site between ACE2 and the RBD of SARS-S and SARS-2S (Gui et al., 2017; Wang et al., 2020; Zhou T. et al., 2020; Xu et al., 2021) does not cover the zinc-binding metallopeptidase domain of ACE2 (Kuba et al., 2010). Therefore, it is theoretically possible to develop a drug that would interfere with the binding between the host ACE2 and the viral RBD without affecting ACE2’s function in converting Ang II to Ang 1–7. Many drug-screening studies check only binding affinity between a candidate drug and human ACE2 (Mathew et al., 2021; Aris et al., 2022). A reasonable drug candidate should bind to the site of interaction between SARS-2S and ACE2, but does not bind to the zinc-binding catalytic site of ACE2.

The same principle of reducing infection without impacting function should be applied not only to host receptors such as ACE2, but also other cofactors such as NRP1 (Cantuti-Castelvetri et al., 2020; Daly et al., 2020) because a proper level of NRP1 protein is essential for cardiovascular and neuronal development (Guo and Vander Kooi, 2015). Overexpression of the gene (Kawasaki et al., 1999), or knock-out of the gene (Kitsukawa et al., 1995) are both lethal in mice.

The function implication of sACE2 remains elusive. The cleavage of ACE2 by ADAM-17 (Haga et al., 2008, 2010; Scheller et al., 2011) is activated in SARS-CoV infection, generating sACE2 (Figure 7A). This could be either a host-mediated protection response or a virus-mediated response to colonize cells not expressing ACE2. If membrane-bound ACE2 mediates viral entry, then cleaving them off membrane would protect the ACE2-expressing cell from infection. However, this hypothesis of host-mediated protection response is contradicted by the observation that the generation of sACE2 enhances infection (Haga et al., 2008, 2010). It is possible that SARS-CoV-2 can bind to both membrane-bound ACE2 and sACE2 which could then bind to membrane proteins such as neuropilin-1 (Cantuti-Castelvetri et al., 2020; Daly et al., 2020), integrins (Nader et al., 2021; Nader and Kerrigan, 2022), or other membrane proteins to anchor SARS-CoV-2 to host cell membrane that do not have ACE2 (Figure 7B). SARS-2S could first bind to sACE2 which then binds to membrane integrin (Figure 7B). This is consistent with the observation that the shedding of ACE2 results in increased uptake of SARS-CoV virions into host cells, and therefore supports the alternative hypothesis of virus-mediated response to colonize host cells that do not express ACE2.

## 4. Predicting mammalian species susceptible to SARS-CoV-2

Many studies have used similarity in ACE2 sequences and sharing of interacting amino acids between ACE2 and the viral RBD to predict vulnerability of other mammalian species to COVID-19 (Damas et al., 2020; Shi et al., 2020; Kruglikov et al., 2021; Wei et al., 2021). The general rationale is that the ACE2 of a mammalian species highly similar to the ACE2 of susceptible species (e.g., human) would serve as a host receptor for SARS-CoV-2 and predispose the species to SARS-CoV-2 infection. SARS-2S can use ACE2 in many species for cell entry (Hossain et al., 2020; Shi et al., 2020; Zhai et al., 2020; Li et al., 2021), including all tested primate species, pangolins, and several carnivorous species.

**Figure 8:**
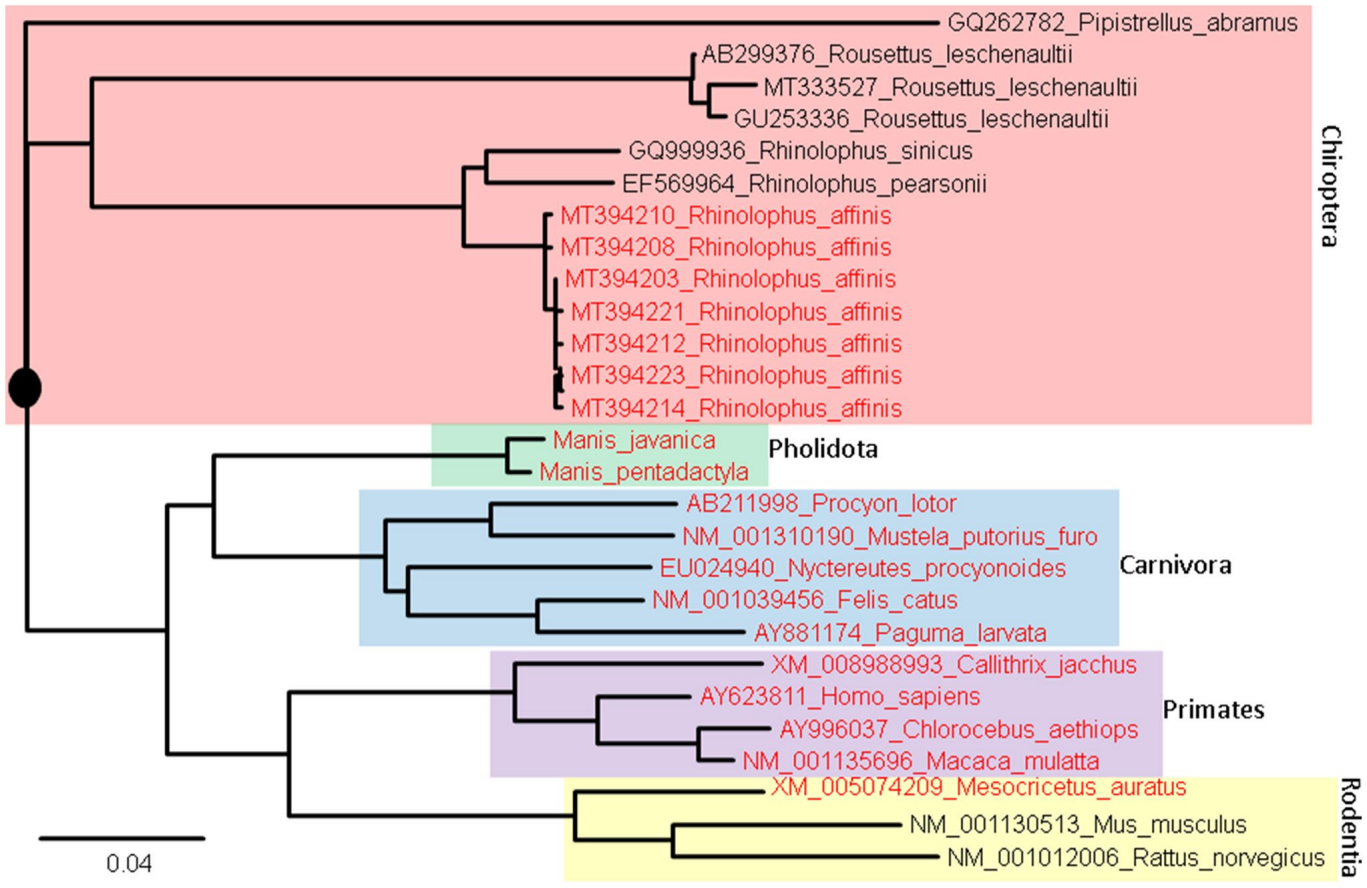
ACE2 Phylogeny of representative mammalian species in Chiroptera (bats), Pholidota (pangolins), Carnivora (felids and canids), Primates, and Rodentia. The protein sequences were aligned by MAFFT (Katoh and Toh, 2008) with the accurate but slow L-INS-i option. The unrooted phylogenetic tree was reconstructed with PhyML (Guindon and Gascuel, 2003), with the empirical LG substitution matrix and optimization of topology, branch lengths and rates. The reconstructed tree is unrooted but is rooted by mid-point. The species in red have been empirically shown to be vulnerable to SARS-CoV-2 infection. They are closer to (have fewer substitutions in ACE2 from) the putative common ancestor (indicated by a black solid circle) than species not vulnerable to SARS-CoV-2 infection.

Two different approaches have been used for the prediction. The first and the simplest index of vulnerability is based on phylogenetic analysis of aligned ACE2 sequences (Figure 8). Species with a short root-to-tip distance have relatively conserved ACE2, and these species, colored in red (Figure 8), tend to be susceptible to SARS-CoV-2 infection. Within rodents, the golden hamster (*Mesocricetus auratus*) can be infected by both SARS-CoV and SARS-CoV-2, and its ACE2 is closer to the putative root than mouse and rat that are not vulnerable unless humanized with human ACE2. Within Chiroptera, *Rhinolophus affinis* is more vulnerable than other bat species (Li et al., 2021) and its ACE2 is closer to the putative root than others (Figure 8). Within primates, human ACE2 is closer to the putative root than other primates, and humans appear to be more vulnerable to COVID-19 infection than other primates.

An ideal receptor protein from a viral perspective is one that is (1) highly expressed in epithelial cell surface of mammalian respiratory or digestive tracts, (2) functionally important so that its expression cannot be readily downregulated by the host in response to the infection, and (3) strongly conserved in evolution so many mammalian species can serve as potential hosts. We have shown previously that ACE2 meets the first two criteria. Figure 8 shows that ACE2 also meets the last criterion.

The second approach for predicting species vulnerability incorporates information from protein structures. The characterization of the structure of SARS-2S (Hoffmann et al., 2020; Walls et al., 2020; Wrapp et al., 2020; Yan et al., 2020), especially those with the SARS-2S trimer in complex with ACE2 (Gui et al., 2017; Wang et al., 2020; Zhou T. et al., 2020; Xu et al., 2021), provides a list of amino acids in physical contact between ACE2 and SARS-2S (Lu et al., 2015; Adhikari et al., 2020; Adhikari and Ching, 2020). The sharing of the interacting amino acids in ACE2 have been used to predict which mammalian species have an ACE2 that can serve as a host receptor for SARS-CoV-2 infection (Shi et al., 2020; Zhai et al., 2020; Kruglikov et al., 2021; Wei et al., 2021).

**Figure 9:**
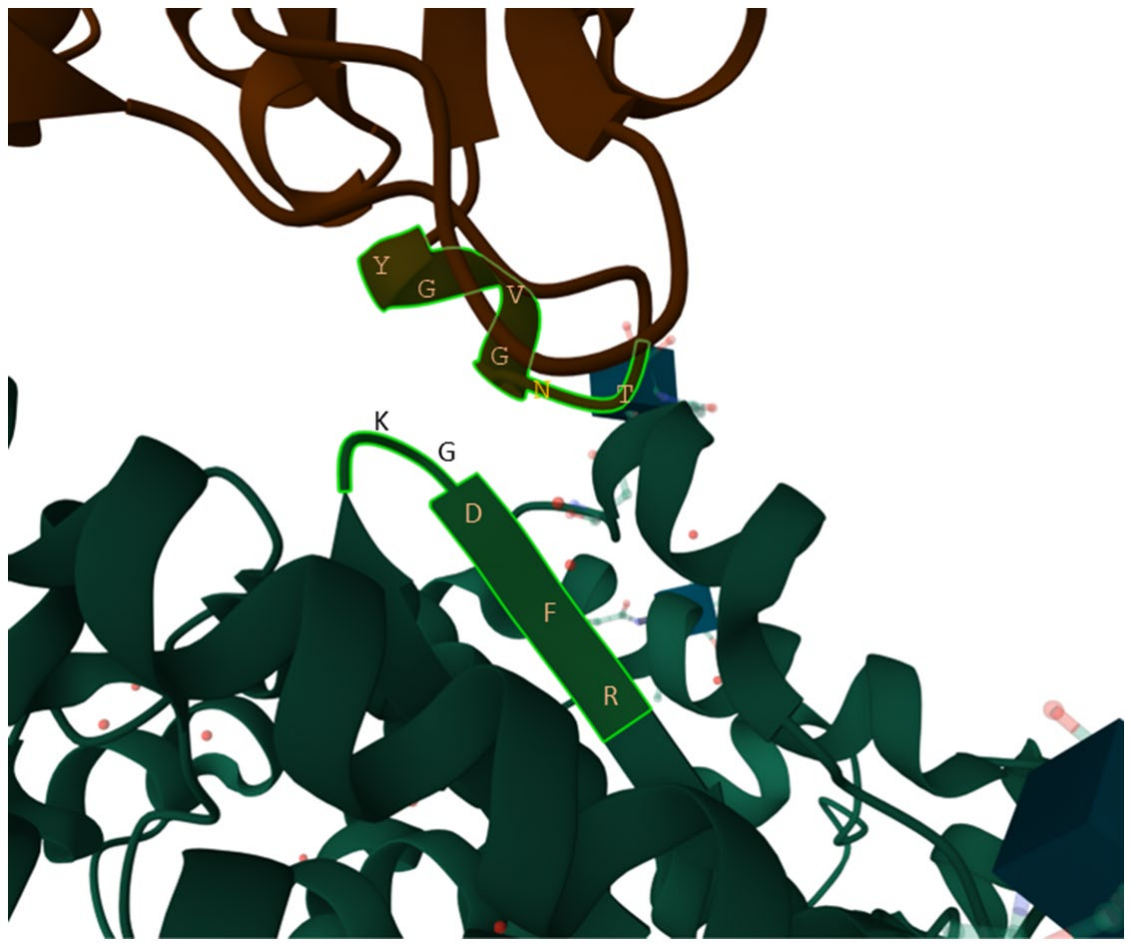
The 353KGDFR357 segment in human ACE2 in close contact with the 500TNGVGY505 segment in SARS-2S, based on structure 6M0J (Lan et al., 2020).

One stretch of five amino acids in human ACE2 (hACE2), 353KGDFR357 (Figure 9), is particularly worth of highlighting because (1) the amino acid composition in the 5-aa motif implies that it is highly hydrophilic and should stay on the surface of the protein, (2) it is in close physical contact with the 500TNGVGY505 segment in SARS-2S (Figure 9), based on the structure 6M0J (Lan et al., 2020), and (3) it is highly conserved, except 354G, across representative species in Carnivora, Artiodactyla and Chiroptera (Wei et al., 2021). A highly conserved hACE2 binding motif means that SARS-2S can not only infect all hACE2, but also ACE2 in a variety of mammalian species. This would generate an unusually large array of potential host species and a consequently large viral reservoir in nature. In contrast, if the interacting motif in hACE2 binding to SARS-2S were highly variable even among different human populations, then SARS-CoV-2 would only be able to infect humans or just a specific genetically homogeneous human population.

Among rodents, the mouse and rat ACE2 that cannot serve as a SARS-2S receptor has 353H. One may infer that an H353K mutation would change the mouse ACE2 to a SARS-2S receptor. Such a mutation has been carried out both in nature and by virologists. The golden hamster (*Mesocricetus auratus*) has 353 K and is susceptible to SARS-CoV-2 infection. Experimental introduction of a H353K mutation into mouse ACE2, i.e., hmACE2.1 in Adams et al. (2021), substantially improved the function of mouse ACE2 as a SARS-2S receptor. There should be more coevolutionary studies between hosts and pathogens.

Prediction of species vulnerability based on ACE2 alone is confounded by many factors. First, successful viral infection involves multiple steps including cell attachment, cell entry, evasion of host immune systems, viral genome replication, transcription, translation, packaging of virions, and cell lysis and viral release. Having a suitable ACE2 receptor represents just one of these steps. For example, pig ACE2 appears to serve as a good receptor for SARS-2S (Li et al., 2021), but SARS-CoV-2 does not infect pigs. Second, an ACE2 in an animal highly similar to human ACE2 may express little ACE2 in respiratory tract. For example, in contrast to humans and other primates, dogs express relatively little ACE2 in respiratory tract but high ACE2 in digestive tract (Naqvi et al., 2019; Zhai et al., 2020), so it is not surprising to find positive rectal swabs but not in pharyngeal swabs in experimental dogs a few days after the inoculation with SARS-CoV-2 (Shi et al., 2020). Thus, a prediction that SARS-CoV-2 would cause respiratory diseases in dogs because dogs have an ACE2 similar to human ACE2 is not quite true because of the low expression of ACE2 in the respiratory tract of dogs.

In summary, host receptor identification and related studies require a multidisciplinary approach involving diverse types of data and integrative data analyses. This review may contribute to the design of training programs for future virologists.

## Author contributions

The author confirms being the sole contributor of this work and has approved it for publication.

## Funding

This research was funded by a Discovery Grant from the Natural Science and Engineering Research Council (NSERC, RGPIN/2018–03878) of Canada. The funders had no role in the design of the study; in the collection, analyses, or interpretation of data; in the writing of the manuscript, or in the decision to publish the results.

## Conflict of interest

The author declares that the research was conducted in the absence of any commercial or financial relationships that could be construed as a potential conflict of interest.

## Publisher’s note

All claims expressed in this article are solely those of the authors and do not necessarily represent those of their affiliated organizations, or those of the publisher, the editors and the reviewers. Any product that may be evaluated in this article, or claim that may be made by its manufacturer, is not guaranteed or endorsed by the publisher.

## References

[ref1] Adams L. E. Dinnon K. H. 3rd Hou Y. J. Sheahan T. P. Heise M. T. Baric R. S. (2021). Critical ACE2 determinants of SARS-CoV-2 and group 2B coronavirus infection and replication. MBio 12:e03149-20. doi: 10.1128/mBio.03149-20, PMID: 33727353 PMC8092278

[ref2] Adhikari P. Ching W. Y. (2020). Amino acid interacting network in the receptor-binding domain of SARS-CoV-2 spike protein. RSC Adv. 10, 39831–39841. doi: 10.1039/D0RA08222H, PMID: 35515388 PMC9057398

[ref3] Adhikari P. Li N. Shin M. Steinmetz N. F. Twarock R. Podgornik R.. (2020). Intra- and intermolecular atomic-scale interactions in the receptor binding domain of SARS-CoV-2 spike protein: implication for ACE2 receptor binding. Physic. Chem. Chem. Physic. 22, 18272–18283. doi: 10.1039/D0CP03145C, PMID: 32756685

[ref4] Aldinger E. E. (1968). Cardiovascular effects of griseofulvin. Circ. Res. 22, 589–593. doi: 10.1161/01.RES.22.5.589, PMID: 5652459

[ref5] Allen J. D. Watanabe Y. Chawla H. Newby M. L. Crispin M. (2021). Subtle influence of ACE2 glycan processing on SARS-CoV-2 recognition. J. Mol. Biol. 433:166762. doi: 10.1016/j.jmb.2020.166762, PMID: 33340519 PMC7744274

[ref6] Amruta N. Engler-Chiurazzi E. B. Murray-Brown I. C. Gressett T. E. Biose I. J. Chastain W. H.. (2021). In vivo protection from SARS-CoV-2 infection by ATN-161 in k18-hACE2 transgenic mice. Life Sci. 284:119881. doi: 10.1016/j.lfs.2021.119881, PMID: 34389403 PMC8352850

[ref7] Andersen K. G. Rambaut A. Lipkin W. I. Holmes E. C. Garry R. F. (2020). The proximal origin of SARS-CoV-2. Nat. Med. 26, 450–452. doi: 10.1038/s41591-020-0820-9, PMID: 32284615 PMC7095063

[ref8] Aris P. Wei Y. Mohamadzadeh M. Xia X. (2022). Griseofulvin: an updated overview of old and current knowledge. Molecules (Basel, Switzerland). 27:7034. doi: 10.3390/molecules27207034, PMID: 36296627 PMC9610072

[ref9] Bao L. Deng W. Huang B. Gao H. Liu J. Ren L.. (2020). The pathogenicity of SARS-CoV-2 in hACE2 transgenic mice. Nature 583, 830–833. doi: 10.1038/s41586-020-2312-y 32380511

[ref10] Bastolla U. Chambers P. Abia D. Garcia-Bermejo M.-L. Fresno M. (2022). Is COVID-19 severity associated with ACE2 degradation? Front. Drug Discov. 1:789710. doi: 10.3389/fddsv.2021.789710

[ref11] Beddingfield B. J. Iwanaga N. Chapagain P. P. Zheng W. Roy C. J. Hu T. Y.. (2021). The integrin binding peptide, ATN-161, as a novel therapy for SARS-CoV-2 infection. JACC Basic Transl Sci. 6, 1–8. doi: 10.1016/j.jacbts.2020.10.003, PMID: 33102950 PMC7566794

[ref12] Belouzard S. Chu V. C. Whittaker G. R. (2009). Activation of the SARS coronavirus spike protein via sequential proteolytic cleavage at two distinct sites. Proc. Natl. Acad. Sci. U. S. A. 106, 5871–5876. doi: 10.1073/pnas.0809524106, PMID: 19321428 PMC2660061

[ref13] Biering S. B. Sarnik S. A. Wang E. Zengel J. R. Leist S. R. Schäfer A.. (2022). Genome-wide bidirectional CRISPR screens identify mucins as host factors modulating SARS-CoV-2 infection. Nat. Genet. 54, 1078–1089. doi: 10.1038/s41588-022-01131-x, PMID: 35879412 PMC9355872

[ref14] Bohan D. van Ert H. Ruggio N. Rogers K. J. Badreddine M. Aguilar Briseño J. A.. (2021). Phosphatidylserine receptors enhance SARS-CoV-2 infection. PLoS Pathog. 17:e1009743. doi: 10.1371/journal.ppat.1009743, PMID: 34797899 PMC8641883

[ref15] Bosso M. Thanaraj T. A. Abu-Farha M. Alanbaei M. Abubaker J. Al-Mulla F. (2020). The two faces of ACE2: the role of ACE2 receptor and its polymorphisms in hypertension and COVID-19. Mol Ther Methods Clin Dev. 18, 321–327. doi: 10.1016/j.omtm.2020.06.017 32665962 PMC7314689

[ref16] Brevini T. Maes M. Webb G. J. John B. V. Fuchs C. D. Buescher G.. (2023). FXR inhibition may protect from SARS-CoV-2 infection by reducing ACE2. Nature. 615, 134–142. doi: 10.1038/s41586-022-05594-0 36470304 PMC9977684

[ref17] Brodowski M. Pierpaoli M. Janik M. Kowalski M. Ficek M. Slepski P.. (2022). Enhanced susceptibility of SARS-CoV-2 spike RBD protein assay targeted by cellular receptors ACE2 and CD147: multivariate data analysis of multisine impedimetric response. Sens Actuat. B Chem. 370:132427. doi: 10.1016/j.snb.2022.132427, PMID: 35911567 PMC9327189

[ref18] Cantuti-Castelvetri L. Ojha R. Pedro L. D. Djannatian M. Franz J. Kuivanen S.. (2020). Neuropilin-1 facilitates SARS-CoV-2 cell entry and infectivity. Science 370, 856–860. doi: 10.1126/science.abd2985, PMID: 33082293 PMC7857391

[ref19] Cavezzi A. Menicagli R. Troiani E. Corrao S. (2022). COVID-19, cation dysmetabolism, sialic acid, CD147, ACE2, viroporins, hepcidin and ferroptosis: a possible unifying hypothesis. F1000Research. 11:102. doi: 10.12688/f1000research.108667.2, PMID: 35340277 PMC8921693

[ref20] Clarke N. E. Fisher M. J. Porter K. E. Lambert D. W. Turner A. J. (2012). Angiotensin converting enzyme (ACE) and ACE2 bind integrins and ACE2 regulates integrin signalling. PLoS One 7:e34747. doi: 10.1371/journal.pone.0034747, PMID: 22523556 PMC3327712

[ref21] Coutard B. Valle C. de Lamballerie X. Canard B. Seidah N. G. Decroly E. (2020). The spike glycoprotein of the new coronavirus 2019-nCoV contains a furin-like cleavage site absent in CoV of the same clade. Antivir. Res. 176:104742. doi: 10.1016/j.antiviral.2020.104742, PMID: 32057769 PMC7114094

[ref22] Dales N. A. Gould A. E. Brown J. A. Calderwood E. F. Guan B. Minor C. A.. (2002). Substrate-based design of the first class of angiotensin-converting enzyme-related carboxypeptidase (ACE2) inhibitors. J. Am. Chem. Soc. 124, 11852–11853. doi: 10.1021/ja0277226, PMID: 12358520

[ref23] Daly J. L. Simonetti B. Klein K. Chen K. E. Williamson M. K. Antón-Plágaro C.. (2020). Neuropilin-1 is a host factor for SARS-CoV-2 infection. Science 370, 861–865. doi: 10.1126/science.abd3072, PMID: 33082294 PMC7612957

[ref24] Damas J. Hughes G. M. Keough K. C. Painter C. A. Persky N. S. Corbo M.. (2020). Broad host range of SARS-CoV-2 predicted by comparative and structural analysis of ACE2 in vertebrates. Proc. Natl. Acad. Sci. 117, 22311–22322. doi: 10.1073/pnas.2010146117, PMID: 32826334 PMC7486773

[ref25] Donoghue M. Hsieh F. Baronas E. Godbout K. Gosselin M. Stagliano N.. (2000). A novel angiotensin-converting enzyme-related carboxypeptidase (ACE2) converts angiotensin I to angiotensin 1-9. Circ. Res. 87, E1–E9. doi: 10.1161/01.RES.87.5.e1, PMID: 10969042

[ref26] Fagerberg L. Hallström B. M. Oksvold P. Kampf C. Djureinovic D. Odeberg J.. (2014). Analysis of the human tissue-specific expression by genome-wide integration of transcriptomics and antibody-based proteomics. Molec Cell. Proteom. 13, 397–406. doi: 10.1074/mcp.M113.035600, PMID: 24309898 PMC3916642

[ref27] Fenizia C. Galbiati S. Vanetti C. Vago R. Clerici M. Tacchetti C.. (2021). SARS-CoV-2 entry: at the crossroads of CD147 and ACE2. Cells 10:1434. doi: 10.3390/cells10061434, PMID: 34201214 PMC8226513

[ref28] Fyhrquist F. Saijonmaa O. (2008). Renin-angiotensin system revisited. J. Intern. Med. 264, 224–236. doi: 10.1111/j.1365-2796.2008.01981.x, PMID: 18793332 PMC7166930

[ref29] Gui M. Song W. Zhou H. Xu J. Chen S. Xiang Y.. (2017). Cryo-electron microscopy structures of the SARS-CoV spike glycoprotein reveal a prerequisite conformational state for receptor binding. Cell Res. 27, 119–129. doi: 10.1038/cr.2016.152, PMID: 28008928 PMC5223232

[ref1001] Guindon S. Gascuel O. (2003). A simple, fast, and accurate algorithm to estimate large phylogenies by maximum likelihood. Syst. Biol. 52, 696–704., PMID: 14530136 10.1080/10635150390235520

[ref30] Guo H. F. Vander Kooi C. W. (2015). Neuropilin functions as an essential cell surface receptor. J. Biol. Chem. 290, 29120–29126. doi: 10.1074/jbc.R115.687327, PMID: 26451046 PMC4705917

[ref31] Haga S. Nagata N. Okamura T. Yamamoto N. Sata T. Yamamoto N.. (2010). TACE antagonists blocking ACE2 shedding caused by the spike protein of SARS-CoV are candidate antiviral compounds. Antivir. Res. 85, 551–555. doi: 10.1016/j.antiviral.2009.12.001, PMID: 19995578 PMC7114272

[ref32] Haga S. Yamamoto N. Nakai-Murakami C. Osawa Y. Tokunaga K. Sata T.. (2008). Modulation of TNF-alpha-converting enzyme by the spike protein of SARS-CoV and ACE2 induces TNF-alpha production and facilitates viral entry. Proc. Natl. Acad. Sci. U. S. A. 105, 7809–7814. doi: 10.1073/pnas.0711241105, PMID: 18490652 PMC2409424

[ref33] Hamming I. Timens W. Bulthuis M. L. Lely A. T. Navis G. van Goor H. (2004). Tissue distribution of ACE2 protein, the functional receptor for SARS coronavirus. A first step in understanding SARS pathogenesis. J. Pathol. 203, 631–637. doi: 10.1002/path.1570, PMID: 15141377 PMC7167720

[ref34] Heurich A. Hofmann-Winkler H. Gierer S. Liepold T. Jahn O. Pöhlmann S. (2014). TMPRSS2 and ADAM17 cleave ACE2 differentially and only proteolysis by TMPRSS2 augments entry driven by the severe acute respiratory syndrome coronavirus spike protein. J. Virol. 88, 1293–1307. doi: 10.1128/JVI.02202-13, PMID: 24227843 PMC3911672

[ref35] Hikmet F. Méar L. Edvinsson Å. Micke P. Uhlén M. Lindskog C. (2020). The protein expression profile of ACE2 in human tissues. Mol. Syst. Biol. 16:e9610. doi: 10.15252/msb.20209610, PMID: 32715618 PMC7383091

[ref36] Hoffmann M. Kleine-Weber H. Pöhlmann S. (2020). A multibasic cleavage site in the spike protein of SARS-CoV-2 is essential for infection of human lung cells. Mol. Cell 78, 779–784.e5. doi: 10.1016/j.molcel.2020.04.022 32362314 PMC7194065

[ref37] Hossain M. G. Javed A. Akter S. Saha S. (2020). SARS-CoV-2 host diversity: an update of natural infections and experimental evidence. J. Microbiol Immunol. Infect 54, 175–181. doi: 10.1016/j.jmii.2020.06.006 32624360 PMC7315156

[ref38] Imai Y. Kuba K. Rao S. Huan Y. Guo F. Guan B.. (2005). Angiotensin-converting enzyme 2 protects from severe acute lung failure. Nature 436, 112–116. doi: 10.1038/nature03712, PMID: 16001071 PMC7094998

[ref39] Inoue Y. Tanaka N. Tanaka Y. Inoue S. Morita K. Zhuang M.. (2007). Clathrin-dependent entry of severe acute respiratory syndrome coronavirus into target cells expressing ACE2 with the cytoplasmic tail deleted. J. Virol. 81, 8722–8729. doi: 10.1128/JVI.00253-07, PMID: 17522231 PMC1951348

[ref40] Kalejaiye T. D. Bhattacharya R. Burt M. A. Travieso T. Okafor A. E. Mou X.. (2022). SARS-CoV-2 employ BSG/CD147 and ACE2 receptors to directly infect human induced pluripotent stem cell-derived kidney podocytes. Front. Cell Develop. Biol. 10:855340. doi: 10.3389/fcell.2022.855340, PMID: 35517495 PMC9065256

[ref1002] Katoh K. Toh H. (2008). Recent developments in the MAFFT multiple sequence alignment program. Brief Bioinform. 9, 286–298. 18372315 10.1093/bib/bbn013

[ref41] Kawasaki T. Kitsukawa T. Bekku Y. Matsuda Y. Sanbo M. Yagi T.. (1999). A requirement for neuropilin-1 in embryonic vessel formation. Development 126, 4895–4902. doi: 10.1242/dev.126.21.4895 10518505

[ref42] Kitsukawa T. Shimono A. Kawakami A. Kondoh H. Fujisawa H. (1995). Overexpression of a membrane protein, neuropilin, in chimeric mice causes anomalies in the cardiovascular system, nervous system and limbs. Development 121, 4309–4318. doi: 10.1242/dev.121.12.4309, PMID: 8575331

[ref43] Kruglikov A. Rakesh M. Wei Y. Xia X. (2021). Applications of protein secondary structure algorithms in SARS-CoV-2 research. J. Proteome Res. 20, 1457–1463. doi: 10.1021/acs.jproteome.0c00734, PMID: 33617253

[ref44] Kuba K. Imai Y. Ohto-Nakanishi T. Penninger J. M. (2010). Trilogy of ACE2: a peptidase in the renin-angiotensin system, a SARS receptor, and a partner for amino acid transporters. Pharmacol. Ther. 128, 119–128. doi: 10.1016/j.pharmthera.2010.06.003, PMID: 20599443 PMC7112678

[ref45] Kuba K. Imai Y. Rao S. Gao H. Guo F. Guan B.. (2005). A crucial role of angiotensin converting enzyme 2 (ACE2) in SARS coronavirus–induced lung injury. Nat. Med. 11, 875–879. doi: 10.1038/nm1267, PMID: 16007097 PMC7095783

[ref46] Kumar R. Baker K. M. Pan J. (2011). “Chapter 9 - activation of the renin-angiotensin system in heart failure” in Heart failure: a companion to Braunwald's heart disease (second edition) ed. Mann D. L. (Philadelphia: W.B. Saunders), 134–151.

[ref47] Kyte J. Doolittle R. F. (1982). A simple method for displaying the hydropathic character of a protein. J. Mol. Biol. 157, 105–132. doi: 10.1016/0022-2836(82)90515-0 7108955

[ref48] Lan J. Ge J. Yu J. Shan S. Zhou H. Fan S.. (2020). Structure of the SARS-CoV-2 spike receptor-binding domain bound to the ACE2 receptor. Nature 581, 215–220. doi: 10.1038/s41586-020-2180-5, PMID: 32225176

[ref49] Li P. Guo R. Liu Y. Zhang Y. Hu J. Ou X.. (2021). The *Rhinolophus affinis* bat ACE2 and multiple animal orthologs are functional receptors for bat coronavirus RaTG13 and SARS-CoV-2. Sci. Bull. (Beijing). 66, 1215–1227. doi: 10.1016/j.scib.2021.01.011, PMID: 33495713 PMC7816560

[ref50] Li M.-Y. Li L. Zhang Y. Wang X.-S. (2020). Expression of the SARS-CoV-2 cell receptor gene ACE2 in a wide variety of human tissues. Infect. Dis. Poverty. 9:45. doi: 10.1186/s40249-020-00662-x 32345362 PMC7186534

[ref51] Li W. Moore M. J. Vasilieva N. Sui J. Wong S. K. Berne M. A.. (2003). Angiotensin-converting enzyme 2 is a functional receptor for the SARS coronavirus. Nature 426, 450–454. doi: 10.1038/nature02145 14647384 PMC7095016

[ref52] Lo Y.-L. Liou G.-G. Lyu J.-H. Hsiao M. Hsu T.-L. Wong C.-H. (2016). Dengue virus infection is through a cooperative interaction between a mannose receptor and CLEC5A on macrophage as a multivalent hetero-complex. PLoS One. 11:e0166474. doi: 10.1371/journal.pone.0166474, PMID: 27832191 PMC5104462

[ref53] Lu G. Wang Q. Gao G. F. (2015). Bat-to-human: spike features determining 'host jump' of coronaviruses SARS-CoV, MERS-CoV, and beyond. Trends Microbiol. 23, 468–478. doi: 10.1016/j.tim.2015.06.003, PMID: 26206723 PMC7125587

[ref54] Mathew S. M. Benslimane F. Althani A. A. Yassine H. M. (2021). Identification of potential natural inhibitors of the receptor-binding domain of the SARS-CoV-2 spike protein using a computational docking approach. Qatar Med. J. 2021:12. doi: 10.5339/qmj.2021.12 34604010 PMC8474837

[ref55] Matsuyama S. Nagata N. Shirato K. Kawase M. Takeda M. Taguchi F. (2010). Efficient activation of the severe acute respiratory syndrome coronavirus spike protein by the transmembrane protease TMPRSS2. J. Virol. 84, 12658–12664. doi: 10.1128/JVI.01542-10, PMID: 20926566 PMC3004351

[ref56] Matsuyama S. Ujike M. Morikawa S. Tashiro M. Taguchi F. (2005). Protease-mediated enhancement of severe acute respiratory syndrome coronavirus infection. Proc. Natl. Acad. Sci. U. S. A. 102, 12543–12547. doi: 10.1073/pnas.0503203102 16116101 PMC1194915

[ref57] Montecino-Rodriguez E. Berent-Maoz B. Dorshkind K. (2013). Causes, consequences, and reversal of immune system aging. J. Clin. Invest. 123, 958–965. doi: 10.1172/JCI64096, PMID: 23454758 PMC3582124

[ref58] Mossel E. C. Wang J. Jeffers S. Edeen K. E. Wang S. Cosgrove G. P.. (2008). SARS-CoV replicates in primary human alveolar type II cell cultures but not in type I-like cells. Virology 372, 127–135. doi: 10.1016/j.virol.2007.09.045, PMID: 18022664 PMC2312501

[ref59] Mu A. Latario C. J. Pickrell L. E. Higgs H. N. (2020). Lysine acetylation of cytoskeletal proteins: emergence of an actin code. J. Cell Biol. 219:e202006151. doi: 10.1083/jcb.202006151 33044556 PMC7555357

[ref60] Nader D. Fletcher N. Curley G. F. Kerrigan S. W. (2021). SARS-CoV-2 uses major endothelial integrin αvβ3 to cause vascular dysregulation in-vitro during COVID-19. PLoS One 16:e0253347. doi: 10.1371/journal.pone.0253347, PMID: 34161337 PMC8221465

[ref61] Nader D. Kerrigan S. W. (2022). Molecular cross-talk between Integrins and Cadherins leads to a loss of vascular barrier integrity during SARS-CoV-2 infection. Viruses 14:891. doi: 10.3390/v14050891, PMID: 35632633 PMC9143673

[ref62] Naqvi S. Godfrey A. K. Hughes J. F. Goodheart M. L. Mitchell R. N. Page D. C. (2019). Conservation, acquisition, and functional impact of sex-biased gene expression in mammals. Science 365:eaaw7317. doi: 10.1126/science.aaw7317, PMID: 31320509 PMC6896219

[ref63] Nguyen L. McCord K. A. Bui D. T. Bouwman K. M. Kitova E. N. Elaish M.. (2022). Sialic acid-containing glycolipids mediate binding and viral entry of SARS-CoV-2. Nat. Chem. Biol. 18, 81–90. doi: 10.1038/s41589-021-00924-1, PMID: 34754101 PMC12434308

[ref64] Nowakowski T. J. Pollen A. A. Di Lullo E. Sandoval-Espinosa C. Bershteyn M. Kriegstein A. R. (2016). Expression analysis highlights AXL as a candidate Zika virus entry receptor in neural stem cells. Cell Stem Cell 18, 591–596. doi: 10.1016/j.stem.2016.03.012, PMID: 27038591 PMC4860115

[ref65] Othman H. Messaoud H. B. Khamessi O. Ben-Mabrouk H. Ghedira K. Bharuthram A.. (2022). SARS-CoV-2 spike protein unlikely to bind to Integrins via the Arg-Gly-asp (RGD) motif of the receptor binding domain: evidence from structural analysis and microscale accelerated molecular dynamics. Front. Mol. Biosci. 9:834857. doi: 10.3389/fmolb.2022.834857, PMID: 35237662 PMC8883519

[ref66] Patel V. B. Clarke N. Wang Z. Fan D. Parajuli N. Basu R.. (2014). Angiotensin II induced proteolytic cleavage of myocardial ACE2 is mediated by TACE/ADAM-17: a positive feedback mechanism in the RAS. J. Mol. Cell. Cardiol. 66, 167–176. doi: 10.1016/j.yjmcc.2013.11.017, PMID: 24332999

[ref67] Peacock T. P. Goldhill D. H. Zhou J. Baillon L. Frise R. Swann O. C.. (2021). The furin cleavage site in the SARS-CoV-2 spike protein is required for transmission in ferrets. Nat. Microbiol. 6, 899–909. doi: 10.1038/s41564-021-00908-w, PMID: 33907312 PMC7619196

[ref68] Plaas M. Seppa K. Gaur N. Kasenõmm P. Plaas M. (2021). Age- and airway disease related gene expression patterns of key SARS-CoV-2 entry factors in human nasal epithelia. Virology 561, 65–68. doi: 10.1016/j.virol.2021.05.012, PMID: 34157565 PMC8214754

[ref69] Plein A. Fantin A. Ruhrberg C. (2014). Neuropilin regulation of angiogenesis, arteriogenesis, and vascular permeability. Microcirculation 21, 315–323. doi: 10.1111/micc.12124, PMID: 24521511 PMC4230468

[ref70] Qiao J. Li W. Bao J. Peng Q. Wen D. Wang J.. (2020). The expression of SARS-CoV-2 receptor ACE2 and CD147, and protease TMPRSS2 in human and mouse brain cells and mouse brain tissues. Biochem. Biophys. Res. Commun. 533, 867–871. doi: 10.1016/j.bbrc.2020.09.042, PMID: 33008593 PMC7489930

[ref71] Robles J. P. Zamora M. Adan-Castro E. Siqueiros-Marquez L. Martinez de la Escalera G. Clapp C. (2022). The spike protein of SARS-CoV-2 induces endothelial inflammation through integrin α5β1 and NF-κB signaling. J. Biol. Chem. 298:101695. doi: 10.1016/j.jbc.2022.101695, PMID: 35143839 PMC8820157

[ref72] Rubin A. A. (1963). Coronary vascular effects of griseofulvin. JAMA 185, 971–972. doi: 10.1001/jama.1963.03060120081033, PMID: 14044234

[ref73] Sadoul K. Wang J. Diagouraga B. Khochbin S. (2011). The tale of protein lysine acetylation in the cytoplasm. J. Biomed. Biotechnol. 2011, 1–15. doi: 10.1155/2011/970382, PMID: 21151618 PMC2997609

[ref74] Scheller J. Chalaris A. Garbers C. Rose-John S. (2011). ADAM17: a molecular switch to control inflammation and tissue regeneration. Trends Immunol. 32, 380–387. doi: 10.1016/j.it.2011.05.005 21752713

[ref75] Sheppard D. (1996). Epithelial integrins. BioEssays 18, 655–660. doi: 10.1002/bies.950180809 8760339

[ref76] Shi J. Wen Z. Zhong G. Yang H. Wang C. Huang B.. (2020). Susceptibility of ferrets, cats, dogs, and other domesticated animals to SARS-coronavirus 2. Science 368, 1016–1020. doi: 10.1126/science.abb7015 32269068 PMC7164390

[ref77] Soler M. J. Wysocki J. Batlle D. (2013). ACE2 alterations in kidney disease. Nephrol. Dial. Transplant. 28, 2687–2697. doi: 10.1093/ndt/gft320, PMID: 23956234 PMC3811059

[ref78] Takada Y. Ye X. Simon S. (2007). The integrins. Genome Biol. 8:215. doi: 10.1186/gb-2007-8-5-215, PMID: 17543136 PMC1929136

[ref79] Teesalu T. Sugahara K. N. Kotamraju V. R. Ruoslahti E. (2009). C-end rule peptides mediate neuropilin-1-dependent cell, vascular, and tissue penetration. Proc. Natl. Acad. Sci. U. S. A. 106, 16157–16162. doi: 10.1073/pnas.0908201106, PMID: 19805273 PMC2752543

[ref80] Tipnis S. R. Hooper N. M. Hyde R. Karran E. Christie G. Turner A. J. (2000). A human homolog of angiotensin-converting enzyme: cloning and functional expression as a captopril-insensitive carboxypeptidase*. J. Biol. Chem. 275, 33238–33243. doi: 10.1074/jbc.M002615200 10924499

[ref81] To K. F. Lo A. W. (2004). Exploring the pathogenesis of severe acute respiratory syndrome (SARS): the tissue distribution of the coronavirus (SARS-CoV) and its putative receptor, angiotensin-converting enzyme 2 (ACE2). J. Pathol. 203, 740–743. doi: 10.1002/path.1597 15221932 PMC7167902

[ref82] To K. F. Tong J. H. Chan P. K. Au F. W. Chim S. S. Chan K. C.. (2004). Tissue and cellular tropism of the coronavirus associated with severe acute respiratory syndrome: an in-situ hybridization study of fatal cases. J. Pathol. 202, 157–163. doi: 10.1002/path.1510, PMID: 14743497 PMC7167900

[ref83] Towler P. Staker B. Prasad S. G. Menon S. Tang J. Parsons T.. (2004). ACE2 X-ray structures reveal a large hinge-bending motion important for inhibitor binding and catalysis. J. Biol. Chem. 279, 17996–18007. doi: 10.1074/jbc.M311191200, PMID: 14754895 PMC7980034

[ref84] Uhlén M. Fagerberg L. Hallström B. M. Lindskog C. Oksvold P. Mardinoglu A.. (2015). Proteomics. Tissue-based map of the human proteome. Science 347:1260419. doi: 10.1126/science.1260419, PMID: 25613900

[ref85] Walls A. C. Park Y. J. Tortorici M. A. Wall A. McGuire A. T. Veesler D. (2020). Structure, function, and antigenicity of the SARS-CoV-2 spike glycoprotein. Cells 181, 281–292. doi: 10.1016/j.cell.2020.02.058 PMC7102599 32155444

[ref86] Wang S. Guo F. Liu K. Wang H. Rao S. Yang P.. (2008). Endocytosis of the receptor-binding domain of SARS-CoV spike protein together with virus receptor ACE2. Virus Res. 136, 8–15. doi: 10.1016/j.virusres.2008.03.004, PMID: 18554741 PMC7114441

[ref87] Wang Q. Zhang Y. Wu L. Niu S. Song C. Zhang Z.. (2020). Structural and functional basis of SARS-CoV-2 entry by using human ACE2. Cells 181, 894–904.e9. doi: 10.1016/j.cell.2020.03.045 PMC7144619 32275855

[ref88] Wei Y. Aris P. Farookhi H. Xia X. (2021). Predicting mammalian species at risk of being infected by SARS-CoV-2 from an ACE2 perspective. Sci. Rep. 11:1702. doi: 10.1038/s41598-41020-80573-x, PMID: 33462320 PMC7814088

[ref89] Wilen C. B. Tilton J. C. Doms R. W. (2012). HIV: cell binding and entry. Cold Spring Harb. Perspect. Med. 2:a006866. doi: 10.1101/cshperspect.a006866, PMID: 22908191 PMC3405824

[ref90] Wrapp D. Wang N. Corbett K. S. Goldsmith J. A. Hsieh C.-L. Abiona O.. (2020). Cryo-EM structure of the 2019-nCoV spike in the prefusion conformation. Science 367, 1260–1263. doi: 10.1126/science.abb2507, PMID: 32075877 PMC7164637

[ref91] Wu F. Zhao S. Yu B. Chen Y. M. Wang W. Song Z. G.. (2020). A new coronavirus associated with human respiratory disease in China. Nature 579, 265–269. doi: 10.1038/s41586-020-2008-3, PMID: 32015508 PMC7094943

[ref92] Wysocki J. Ye M. Soler M. J. Gurley S. B. Xiao H. D. Bernstein K. E.. (2006). ACE and ACE2 activity in diabetic mice. Diabetes 55, 2132–2139. doi: 10.2337/db06-0033 16804085

[ref93] Xia X. (2018a). “Bioinformatics and translation elongation” in Bioinformatics and the cell: Modern computational approaches in genomics, proteomics and transcriptomics, ed. Xia X. (Switzerland: Springer, Cham), 197–238.

[ref94] Xia X. (2018b). DAMBE7: new and improved tools for data analysis in molecular biology and evolution. Mol. Biol. Evol. 35, 1550–1552. doi: 10.1093/molbev/msy073, PMID: 29669107 PMC5967572

[ref95] Xia X. (2021). Domains and functions of spike protein in SARS-COV-2 in the context of vaccine design. Viruses 13:109. doi: 10.3390/v13010109, PMID: 33466921 PMC7829931

[ref96] Xu C. Wang Y. Liu C. Zhang C. Han W. Hong X.. (2021). Conformational dynamics of SARS-CoV-2 trimeric spike glycoprotein in complex with receptor ACE2 revealed by cryo-EM. Science Advances 7:abe5575. doi: 10.1126/sciadv.abe5575 PMC7775788 33277323

[ref97] Xu H. Zhong L. Deng J. Peng J. Dan H. Zeng X.. (2020). High expression of ACE2 receptor of 2019-nCoV on the epithelial cells of oral mucosa. Int. J. Oral Sci. 12:8. doi: 10.1038/s41368-020-0074-x, PMID: 32094336 PMC7039956

[ref98] Yan R. Zhang Y. Li Y. Xia L. Guo Y. Zhou Q. (2020). Structural basis for the recognition of SARS-CoV-2 by full-length human ACE2. Science 367, 1444–1448. doi: 10.1126/science.abb2762, PMID: 32132184 PMC7164635

[ref99] Yan Y. Zhou A. Carrell R. W. Read R. J. (2019). Structural basis for the specificity of renin-mediated angiotensinogen cleavage. J. Biol. Chem. 294, 2353–2364. doi: 10.1074/jbc.RA118.006608, PMID: 30563843 PMC6378967

[ref100] Yang C. Zhang Y. Zeng X. Chen H. Chen Y. Yang D.. (2021). Kidney injury molecule-1 is a potential receptor for SARS-CoV-2. J. Mol. Cell Biol. 13, 185–196. doi: 10.1093/jmcb/mjab003, PMID: 33493263 PMC7928767

[ref101] Zech F. Schniertshauer D. Jung C. Herrmann A. Cordsmeier A. Xie Q.. (2021). Spike residue 403 affects binding of coronavirus spikes to human ACE2. Nat. Commun. 12:6855. doi: 10.1038/s41467-021-27180-0, PMID: 34824253 PMC8617078

[ref102] Zhai X. Sun J. Yan Z. Zhang J. Zhao J. Zhao Z.. (2020). Comparison of severe acute respiratory syndrome coronavirus 2 spike protein binding to ACE2 receptors from human, pets, farm animals, and putative intermediate hosts. J. Virol. 94:e00831-20. doi: 10.1128/JVI.00831-20, PMID: 32404529 PMC7375388

[ref103] Zhao Y. Zhao Z. Wang Y. Zhou Y. Ma Y. Zuo W. (2020). Single-cell RNA expression profiling of ACE2, the receptor of SARS-CoV-2. Am. J. Respir. Crit. Care Med. 202, 756–759. doi: 10.1164/rccm.202001-0179LE, PMID: 32663409 PMC7462411

[ref104] Zheng M. (2022). ACE2 and COVID-19 susceptibility and severity. Aging Dis. 13, 360–372. doi: 10.14336/AD.2021.0805, PMID: 35371596 PMC8947832

[ref105] Zhou T. Tsybovsky Y. Gorman J. Rapp M. Cerutti G. Chuang G. Y.. (2020). Cryo-EM structures of SARS-CoV-2 spike without and with ACE2 reveal a pH-dependent switch to mediate endosomal positioning of receptor-binding domains. Cell Host Microbe 28, 867–879.e5. doi: 10.1016/j.chom.2020.11.004, PMID: 33271067 PMC7670890

[ref106] Zhou P. Yang X.-L. Wang X.-G. Hu B. Zhang L. Zhang W.. (2020). A pneumonia outbreak associated with a new coronavirus of probable bat origin. Nature 579, 270–273. doi: 10.1038/s41586-020-2012-7, PMID: 32015507 PMC7095418

